# Spatial Learning and Action Planning in a Prefrontal Cortical Network Model

**DOI:** 10.1371/journal.pcbi.1002045

**Published:** 2011-05-19

**Authors:** Louis-Emmanuel Martinet, Denis Sheynikhovich, Karim Benchenane, Angelo Arleo

**Affiliations:** Laboratory of Neurobiology of Adaptive Processes, UMR 7102, CNRS - UPMC Univ P6, Paris, France; Indiana University, United States of America

## Abstract

The interplay between hippocampus and prefrontal cortex (PFC) is fundamental to spatial cognition. Complementing hippocampal place coding, prefrontal representations provide more abstract and hierarchically organized memories suitable for decision making. We model a prefrontal network mediating distributed information processing for spatial learning and action planning. Specific connectivity and synaptic adaptation principles shape the recurrent dynamics of the network arranged in cortical minicolumns. We show how the PFC columnar organization is suitable for learning sparse topological-metrical representations from redundant hippocampal inputs. The recurrent nature of the network supports multilevel spatial processing, allowing structural features of the environment to be encoded. An activation diffusion mechanism spreads the neural activity through the column population leading to trajectory planning. The model provides a functional framework for interpreting the activity of PFC neurons recorded during navigation tasks. We illustrate the link from single unit activity to behavioral responses. The results suggest plausible neural mechanisms subserving the cognitive “insight” capability originally attributed to rodents by Tolman & Honzik. Our time course analysis of neural responses shows how the interaction between hippocampus and PFC can yield the encoding of manifold information pertinent to spatial planning, including prospective coding and distance-to-goal correlates.

## Author Summary

We study spatial cognition, a high-level brain function based upon the ability to elaborate mental representations of the environment supporting goal-oriented navigation. Spatial cognition involves parallel information processing across a distributed network of interrelated brain regions. Depending on the complexity of the spatial navigation task, different neural circuits may be primarily involved, corresponding to different behavioral strategies. Navigation planning, one of the most flexible strategies, is based on the ability to prospectively evaluate alternative sequences of actions in order to infer optimal trajectories to a goal. The hippocampal formation and the prefrontal cortex are two neural substrates likely involved in navigation planning. We adopt a computational modeling approach to show how the interactions between these two brain areas may lead to learning of topological representations suitable to mediate action planning. Our model suggests plausible neural mechanisms subserving the cognitive spatial capabilities attributed to rodents. We provide a functional framework for interpreting the activity of prefrontal and hippocampal neurons recorded during navigation tasks. Akin to integrative neuroscience approaches, we illustrate the link from single unit activity to behavioral responses while solving spatial learning tasks.

## Introduction

Spatial cognition requires long-term neural representations of the spatiotemporal properties of the environment [1]. These representations are encoded in terms of multimodal descriptions of the animal-environment interaction during active exploration. Exploiting these contextual representations (e.g. through reward-based learning) can produce goal-oriented behavior under different environmental conditions and across subsequent visits to the environment. The complexity of the learned neural representations has to be adapted to the complexity of the spatial task and, consequently, to the flexibility of the navigation strategies used to solve it [2], [3]. Spatial navigation *planning* —defined here as the ability to mentally evaluate alternative sequences of actions to infer optimal trajectories to a goal— is among the most flexible navigation strategies [3]. It can enable animals to solve hidden-goal tasks even in the presence of dynamically blocked pathways (e.g. *detour* navigation tasks, [4]). Experimental and theoretical works have identified three main types of representations suitable for spatial navigation planning, namely route-based, topological, and metrical maps [2], [3], [5]–[7]. Route-based representations encode sequences of place-action-place associations independently from each other, which does not guarantee optimal goal-oriented behavior (e.g. in terms of capability of either finding the shortest pathway or solving detour tasks). Topological maps merge routes into a common goal-independent representation that can be understood as a graph whose nodes and edges encode spatial locations and their connectivity relations, respectively [2]. Topological maps provide compact representations that can generate coarse spatial codes suitable to support navigation planning in complex environments. Metrics-based maps go beyond pure topology in the sense they embed the metrical relations between environmental places and/or cues —i.e. their distances and angles— within an allocentric (i.e. world centered) reference frame [5]. Here, we model a spatial memory system that primarily learns topological maps. In addition, the resultant representation also encodes directional-related information, allowing some geometrical regularities of the environment to be captured. The encoding of metric information favors the computation of novel pathways (e.g. shortcuts) even through unvisited regions of the environment. In contrast to the qualitative but operational space code provided by topological maps, metrical representations form more precise descriptions of the environment that are available only at specific locations until the environment has been extensively explored [5]. However, purely metric representations are prone to errors affecting distance and angle estimations (e.g. path integration [8]). Behavioral and neurophysiological data suggest the coexistence of multiple memory systems that, by being instrumental in the encoding of routes, topological maps and metrical information, cooperate to subserve goal-oriented navigation planning [9].

An important question is how these representations can be encoded by neural populations within the brain. Similar to other high-level functions, spatial cognition involves parallel information processing mediated by a network of brain structures that interact to promote effective spatial behavior [3], [9]–[11]. An extensive body of experimental work has investigated the neural bases of spatial cognition, and a significant amount of evidence points towards a prominent role of the hippocampal formation [12]. This limbic region has been thought to mediate spatial learning functions ever since location-selective neurons —namely hippocampal *place cells* [1], and entorhinal *grid cells* [13]— and orientation-selective neurons —namely *head-direction cells* [14]— were observed by means of electrophysiological recordings from freely moving rats. Yet, the role of the hippocampal formation in goal representation and reward-dependent navigation planning remains unclear [15]. On the one hand, the hippocampus has been proposed to encode topological-like representations suitable for action sequence learning [16] (see [15] for a review of models). This hypothesis mainly relies on the recurrent dynamics generated by the CA3 collaterals of the hippocampus [17]. On the other hand, the hippocampal space code is likely to be highly redundant and distributed [18], which does not seem adequate for learning compact topological representations of high-dimensional spatial contexts. Also, the experimental evidence for high-level spatial representations mediated by a network of neocortical areas (e.g. the posterior parietal cortex [19] and the prefrontal cortex [20]) suggests the existence of an extra-hippocampal action planning system shared among multiple brain regions [21], [22]. The model presented here relies on the hypothesis of a distributed spatial cognition system in which the hippocampal formation would contribute to navigation planning by conveying redundant spatial representations to higher associative areas, and a cortical network would elaborate more compact representations of the spatial context —accounting for motivation-dependent memories, action cost/risk constraints, and temporal sequences of goal-directed behavioral responses [23].

Among the cortical areas involved in map building and action planning, the prefrontal cortex (PFC) is likely to play a central role, as suggested by anatomical PFC lesion studies showing impaired navigation planning in rats [24], [25] and neuroimaging studies [26],[27]. Also, the anatomo-functional properties of the PFC seem appropriate to encode multimodal contextual memories that are not merely based on spatial correlates. The PFC receives direct projections from sub-cortical structures (e.g. the hippocampus [28], the thalamus [29], the amygdala [30] and the ventral tegmental area [31]), and indirect connections from the basal ganglia through the basal ganglia - thalamocortical loops [32]. These projections convey multidimensional information onto the PFC, including (but not limited to) emotional and motivational inputs [33], reward-dependent modulation [34], and action-related signals [32]. The PFC seems then well suited to *(i)* process manifold spatial information [35], *(ii)* encode the motivational values associated to spatiotemporal events [15], and *(iii)* perform supra-modal decision making [36], [37]. Also, the PFC may be involved in integrating events in the temporal domain at multiple time scales [38]. Indeed, its recurrent dynamics, regulated by the modulatory action of dopaminergic afferents, may maintain patterns of activity over long time scales [39]. Finally, the PFC is likely to be critical to detecting cross-temporal contingencies, which is relevant to the temporal organization of behavioral responses, and to the encoding of retrospective and prospective memories [38].

This article presents a neurocomputational model of the PFC columnar organization [40] and focuses on its possible role in spatial navigation planning. The cortical column model generates compact topological maps from afferent redundant spatial representations encoded by the hippocampal place cell activity patterns as modeled by Sheynikhovich et al. [41]. The model exploits the multimodal coding property offered by the possibility to refine the cortical architecture by adding a sublevel to the column, i.e. the minicolumn. It also exploits the recurrent nature of the columnar organization to learn multilevel topological maps accounting for structural regularities of the environment (such as maze alleys and arms). It shows how specific connectivity principles regulated by unsupervised Hebbian mechanisms for synaptic adaptation can mediate the learning of topological neural representations in the PFC. Then, the model uses the underlying topological maps to plan goal-directed pathways through a neural implementation of a simple breadth-first graph search mechanism called activation diffusion or spreading activation [42]–[44]. The activation diffusion process is based on the propagation of a reward-dependent signal from the goal state through the entire topological network. This propagation process enables the system to generate action sequences (i.e. trajectories) from the current position towards the goal. We show how the modeled anatomo-functional interaction between the hippocampal formation and the prefrontal cortex can enable simulated rats to learn detour navigation tasks such as Tolman & Honzik's task [4]. The model presented here aims at shedding some light on the link between single-cell activity and behavioral responses. We perform a set of statistical and information theoretical analyses to characterize the encoding properties of hippocampal and PFC neuronal activity —in terms of both main correlates (e.g. location, distance-to-goal, and prospective coding) and functional time course changes. We interpret and validate the results of these analyses against available experimental data (e.g. extracellular electrophysiological recordings of PFC units).

## Materials and Methods

### Cortical column model for spatial learning and navigation planning

Cortical maps consist of local circuits —i.e. the cortical columns [40]— that share common features in sensory, motor and associative areas, and thus reflect the modular nature of cortical organization and function [45]. Cortical columns can be divided in six main layers including: layer I, which mostly contains axons and dendrites; layers II-III, called *supragranular* layers, which are specialized in cortico-cortical connections to both adjacent and distant cortical zones; layer IV, which receives sensory inputs from sub-cortical structures (mainly the thalamus) or from columns of cortical areas involved in earlier stages of sensory processing; and layers V–VI, called *infragranular* layers, which send outputs to sub-cortical brain areas (e.g. to the striatum and the thalamus) regulating the ascending information flow through feedback connections. According to the cytoarchitectonic properties of the rat medial PFC [32], no layer IV is considered in the model of cortical column described henceforth. Neuroanatomical findings (see [45] for a review; see [46], [47] for anatomical data on rat PFC) suggest that columns can be further divided into several *minicolumns*, each of which consists of a population of interconnected neurons [48]. Thus, a column can be seen as an ensemble of interrelated minicolumns receiving inputs from cortical and sub-cortical areas. It processes these afferent signals and projects the responses both within and outside the cortical network. This twofold columnar organization has been suggested to subserve efficient computation and information processing [45], [49]. Several models have been proposed to study the cortical columnar architecture, from early theories on cortical organization [50]–[52] to recent computational approaches (e.g. the blue brain project [53]). These models either provide a detailed description of the intrinsic organization of the column in relation to cytological properties and cell differentiation or focus on purely functional aspects of columnar operations.

**Figure 1:**
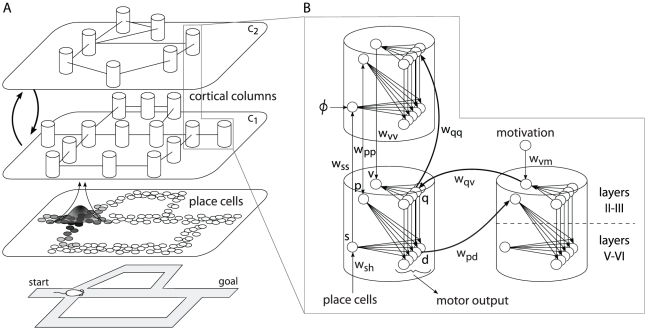
Overview of the model architecture and connectivity. (**A**) Model hippocampal place (HP) cells are selective to allocentrically-encoded positions. The prefrontal cortex (PFC) columnar network takes HP cell activities as input to learn a sparse state-action code 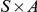 reflecting the topological organization of the environment. The model employs recurrent excitatory collaterals between minicolumns of two subpopulations (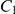 and 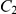) to implement multilevel spatial processing capturing morphological regularities of the environment. (**B**) Each model column uses three units 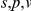 and a population of minicolumns, each of which is composed of two units 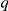 and 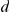. Neurons 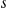 receive inputs from HP cells through 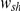 synapses to encode spatial locations. Forward and backward associations between locations are encoded by 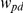 and 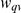 connections, respectively, so that the minicolumn corresponding to the execution of an action in a given place is linked to the place visited after movement. The model uses a motivational signal conveyed by 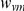 synapses to encode goal information. The population of neurons 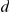 projects to motor output, where a winner-take-all competition takes place to select actions locally. Collateral projections between columns (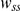, 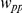, 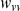 and 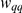) together with a proprioceptive signal 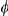 allow the model to implement multilevel spatial processing.

The approach presented here attempts to relate the columnar organization to decision making and behavioral responses using a highly simplified neural architecture which does not account for cell diversity and biophysical properties of PFC neurons. Fig. 1A shows an overview of the model architecture based on this notion of cortical column organization. As aforementioned, the underlying hypothesis is that the PFC network may mediate a sparsification of the hippocampal place (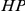) representation to encode topological maps and subserve goal-directed action planning. The model exploits the anatomical excitatory projections from hippocampus to PFC [28] to convey the redundant 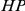 state-space representation 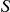 to the columnar PFC network, where a sparse state-action code 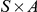 is learned. Within a column, each minicolumn becomes selective to a specific state-action pair 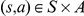, with actions 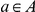 representing allocentric motion directions to perform transitions between two states 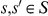. Each column is thus composed by a population of minicolumns that represent all the state-action pairs 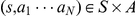 experienced by the animal at a location 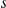. This architecture is consistent with data showing that minicolumns inside a column have similar selectivity properties [54] and that some PFC units encode purely cue information while others respond to cue-response associations [55].

The model employs the excitatory collaterals between minicolumns [45], [56] to learn multilevel topological representations. Egocentric self-motion information (provided by proprioceptive inputs) biases the selectivity properties of a subpopulation of columns to capture morphological regularities of the environment. Unsupervised learning also modulates the recurrent projections between minicolumns to form forward and reverse associations between states. During planning, the spreading of a reward signal from the column selective for the goal through the entire network mediates the retrieval of goal-directed pathways. Then, a local competition between minicolumns allows the most appropriate goal-directed action to be inferred.

The following sections provide a functional description of the model columnar structure, connectivity and input-output functional properties. A more comprehensive account –including equations, parameter settings and explanatory figures– can be found in Supplementary Text S1.

#### Encoding topological maps by a network of columns

Every column in the model (Fig. 1B) has a highly simplified structure consisting of three units 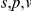 and of a population of minicolumns, each of which is composed of two units 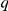 and 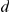. The activity of each of these units (see Supplementary Text S1) represents the mean firing rate of a population of pyramidal neurons either in supragranular layers II–III (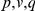 units) or in infragranular layers V–VI (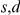 units).

As exploration proceeds, 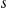 neurons become selective to spatial locations —due to the driving input from hippocampal place cells (Fig. 1B). In the model, hippocampal representations integrate visual and self-motion cues, and result in populations of Gaussian-shaped place fields (see [41], [57], [58] for detailed accounts). During spatial learning, at each location visited by the simulated animal, an unsupervised Hebbian scheme reinforces the projections from the subset of active place cells to the most active 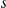 unit (see Supplementary Text S1). As a result, the population activity of 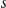 units tends to encode more compact state-space representations than hippocampal place fields. Note that the unsupervised learning scheme begins to reinforce afferent connections to 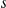 units only when the place field representation has become stable (i.e. every place is encoded by a sub-population of place cells, see Supplementary Text S1 Sec. Spatial learning: encoding topological representations).

Within each column one neuron 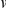 encodes goal information related to a specific state, whereas neurons 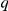 encode the relation between actions and goal. Neurones 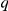 and 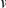 back-propagate the goal signal through the cortical network and their discharge correlates to the distance to the goal. Neurones 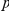 forward-propagate the selected path signal (i.e. the planned trajectory) from a given position towards the goal. Neurones 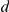 integrate spatial and reward-related information and compete for local action selection. Their activity triggers a motor command tuned to a specific allocentric motion direction. Inter- and intra-column connectivity (Fig. 1B, see also Supplementary Text S1) involves plastic and non-plastic projections, respectively, whose synaptic efficacies are modeled as scalar weight matrices 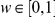. Plastic synapses are randomly initialized to low efficacy values within 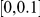, i.e. the cortical network starts with weak interconnectivity. As the simulated animal explores the environment, plastic projections are modified through unsupervised Hebbian learning to encode either states or forward and reverse associations between adjacent states (i.e. environment topology). For instance, whenever the simulated rat moves from one place to another, collateral projections 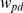 and 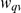 (Fig. 1B) are updated to reflect to connectedness between the two places.

#### Navigation planning through activation diffusion of reward-dependent signals

The simulated animal behaves to either improve its representation or follow known goal-directed pathways (see Supplementary Text S1). This exploration-exploitation trade off is governed by a simple stochastic policy [58]. During exploration, motivation-dependent signals modulate the activity of neurons 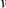 in layer II-III of the model (Fig. 1B), which allows specific columns to become selective to reward states. The reward-related signal transmitted by 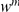 projections simulates a physiological drive mediated by either dopaminergic neurons in the ventral tegmental area [34] or the amygdala [33], both sending synapses to the prefrontal cortex [32]. An activation diffusion process [52] supports the exploitation of topological information to retrieve optimal trajectories to the goal. The motivation signal elicits the activity of the 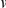 neuron in the column corresponding to the goal location. This reward-based activity is then back-propagated through reverse associations mediated by the lateral projections 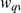 (Fig. 1B). When the back-propagated goal signal reaches the column selective for the current position, the coincidence of 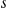 and 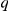 activity triggers the discharge of neurons 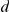. The 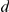 activation, in turns, activates the forward propagation of a goal-directed signal through projections 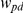. Since 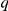 neurons are already active, successive discharges of 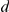 and 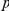 neurons allow the path signal to spread forward to the goal column. A competitive winner-take-all scheme, which locally selects the motor action 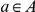 associated to the most active neuron 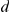, reads out goal-directed trajectories.

It is worth mentioning that projections 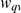 attenuate the back-propagating activity such that the smaller is the number of synaptic relays, the stronger is the goal signal received by the 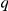 neurons of the column corresponding to the current location. Thus, the activation diffusion mechanism produces an exponential decrease of the intensity of the goal signal that propagates along the network of columns. Since the receptive fields of the model columns tend to be evenly distributed over the environment, the intensity of the goal signal at a given place does correlate with the distance to the rewarding location. In other words, the columnar network encodes goal-related metrical information allowing the shortest pathway to the target to be selected.

#### Recurrent cortical processing for multilevel topological mapping

The model can learn hierarchical state-space representations by employing recurrent projections between columns [45], [56]. As shown in Fig. 1 (but see Supplementary Text S1 for more details), this multistage processing can simply be understood in terms of the interaction between two subpopulations of cortical columns. The first population 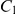 receives and processes direct spatial inputs from the hippocampus. The second population 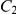 receives already processed state information from neurons 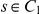, but the dynamics of the neurons 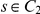 is also modified by a putative proprioceptive signal 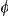, modulating their electroresponsiveness and the synaptic plasticity between neurons 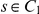 and 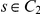. This 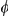 signal encodes the probability of sharp motion direction changes at a particular location. Thus, while moving along a corridor for instance, the signal remains constant and allows for the potentiation of synapses between multiple neurons 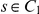 and one unit 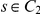. At a turning point, the signal 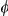 changes its value which may result in the recruitment of a new 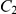 column (see Supplementary Text S1 for implementation details). As a consequence the selectivity of neurons 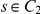 accounts for the presence of structural features of the environment such as alleys and corridors. The spatial resolution of the resultant multilevel representation can then adapt to the structural complexity of the maze.

The 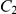 columnar network, which is learned similarly to the 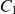 network, also supports the activation diffusion mechanism to plan goal-directed trajectories (Supplementary Text S1). After learning, collateral projections 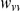 and 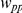 allow 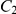 to modulate the activity of neurons 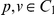 during planning (Fig. 1B).

### Spatial learning tasks and statistical analyses

We demonstrate the ability of the model to learn topological representations and plan goal-oriented trajectories by considering a navigation task: the Tolman & Honzik's *detour* task. The behavioral responses of simulated rats are constraint by intersecting alleys, which, in contrast to open field mazes, generate clear decision points and permit dynamic blocking of goal-directed pathways.

#### Tolman & Honzik's detour task

**Figure 2:**
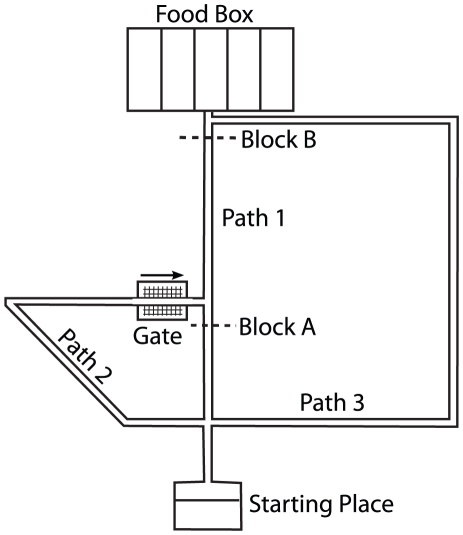
Spatial navigation tasks used to test the capability of inferring detours. The Tolman & Honzik's maze (adapted from [4]) consists of three pathways (Path 1, Path 2 and Path 3) with different lengths. The original maze fits approximately within a rectangle of 1.20×1.55 m. Two blocks can be introduced to prevent animals from navigating through Path 1 (Block A) or both Path 1 and Path 2 (Block B). The gate near the second intersection prevents rats from going from right to left.

The classical Tolman & Honzik's maze (Fig. 2) consisted of three narrow alleys of different lengths (Paths 1, 2, and 3) guiding the animals from a starting location to a feeder location. Tolman & Honzik's experiment aimed at corroborating the hypothesis that rodents, while undergoing a navigation task, can predict the outcomes of alternative goal-directed trajectories in the presence of dynamically blocked pathways. We implemented Tolman & Honzik's experimental setup within the Webotssimulator. The latter provided a realistic three dimensional environment where simulated rats could process visual and proximity information (provided by whisker-like sensors), as well as self-motion (proprioceptive-like) signals. Simulated rats were moving at constant speed (15 cm/s). We ran a series of numerical simulations to emulate the experimental protocol originally designed by Tolman & Honzik:

The *training period* lasted 168 trials (that correspond to 14 days with 12 trials per day), during which the simulated animals could explore the maze to elaborate topological representations and learn navigation policies. In the following, we refer to 12 training trials as a “day” of simulation.

• *Day 1*. A series of 3 *forced runs* was carried out in which the simulated rats were forced to go through P1, P2, and P3 successively. Then, during the remaining 9 trials, the subjects were allowed to explore the maze freely. At the end of Day 1, a preference for P1 was expected to be already established [4].

• *Day 2 to 14*. On each trial, a block was introduced at location A (Block A, Fig. 2) to induce a choice between P2 and P3. Entrances to P2 and P3 were also blocked in order to force the animals to go first to Block A. When the simulated rats reached block A and returned back to the first intersection, doors were removed and subjects had to decide between P2 and P3. Every day, 10 runs with a block at A were mixed with 2 non-successive free runs to maintain the preference for P1.

The *probe test* lasted 7 trials (Day 15) with a block at location B (Block B, Fig. 2) to interrupt the portion of pathway shared by P1 and P2. Animals were forced to decide between P2 and P3 when returning to the first intersection point. Both training and probe trials ended when the simulated animal reached the goal, i.e. when it crossed the entrance to the food box.

To assess the invariance of the model performance with respect to the size of the environment, we implemented the above experimental protocol for two different maze scales, 1∶1 and 4∶1. We took the dimensions of the simulated mazes so as to maintain the proportions of Tolman & Honzik's setup.

We employed a population of 40 simulated rats for each experimental protocol. We quantified the statistical significance of the results by means of an ANOVA analysis (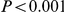 was considered significant).

#### Statistical analysis of neural activities

We analyzed the activity patterns of simulated neurons in relationship to electrophysiological data. This study aimed at elucidating the link between cell activity and behavior and it stressed the importance of relating the time course profile of single cell discharges to decision-related behavioral responses. This was done by: (i) characterizing the spatial selectivity properties of single cell types; (ii) comparing the density —and other correlated measures such as sparseness and redundancy— of the spatial population codes learned by simulated animals (we recall that one of the aims of the cortical column model was to build spatial codes less redundant than hippocampal place field representations); (iii) differentiating the coding properties of purely reward-related neurons (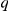 and 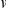 populations) vs. purely spatial units (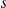 population); (iv) quantifying and comparing the reliability of neural spatial representations (both at level of single cell and population code) in terms of information content —i.e. how much can we infer about either the animal's position or a particular phase of the task by observing neural responses only? See supplementary Text S2, for details on the statistical measures and parameters employed to perform data analyses.

Besides relating our simulation results to literature experimental data, we studied the consistency between model neural responses and a set of PFC electrophysiological recordings from navigating rats. In these experiments —carried out at S.I. Wiener's laboratory; see detailed methods in [59], [60]— extracellular recordings were performed from medial PFC pyramidal cells of Long-Evans rats solving a spatial memory task. The analysis presented here investigated whether the coding properties of all types of neurons in the cortical network model could actually be observed in the PFC during spatial learning.

## Results

### Spatial behavior in Tolman & Honzik's detour task

**Figure 3:**
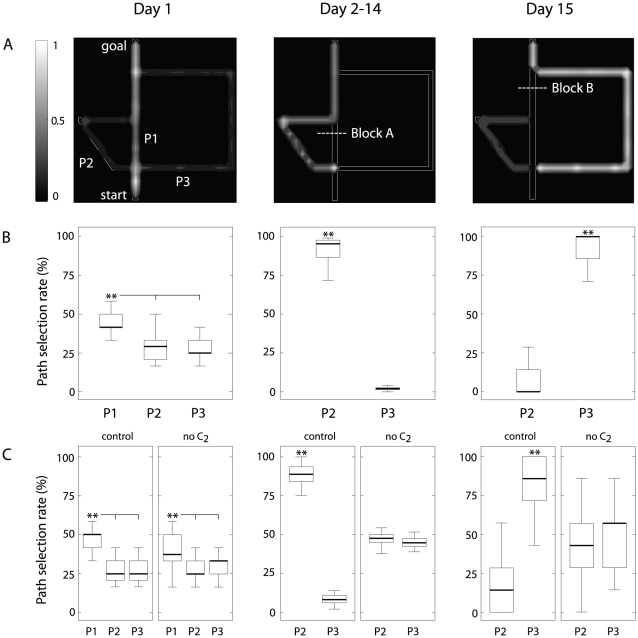
Spatial behavior performance in the Tolman & Honzik's detour task. Simulation results. Day 1: left column; Day 2–14: central column; Day 15: right column. (**A**) Occupancy grids representing path selection results qualitatively. (**B**) Mean path selection rate (averaged over 40 simulated animals) in the 1∶1 scale version of the maze. Note that similar to Tolman & Honzik [4] we ignored P1 in Day 2–14 and Day 15 analyses because blocked. (**C**) Performance of “control” vs. “no 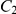
” animals in the 4∶1 version of Tolman & Honzik's maze.

We first examined the behavioral responses of 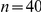 simulated animals solving the 1∶1 version of Tolman & Honzik's task (see Sec. sec:tolmantask and Fig. 2 for details on the experimental apparatus and protocol). The qualitative and quantitative results shown on Figs. 3A and B, respectively, demonstrate that the model reproduced the behavioral observations originally reported by Tolman & Honzik [4].

During the first 12 training trials (*Day 1*) the simulated animals learned the topology of the maze and planned their navigation trajectories in the absence of blocks A and B. Similar to Tolman & Honzik's findings, the model selected the shortest pathway P1 significantly more than alternative paths P2 and P3 (ANOVA, 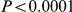; Figs. 3A,B left column).

During the following 156 training trials (*Days 2–14*), a block at location A forced the animals to update their topological maps dynamically, and plan a detour to the goal. The results reported by Tolman & Honzik provided strong evidence for a preference for the shortest *detour* path P2. Consistently, we observed a significantly larger number of transits through P2 compared to P3 (ANOVA, 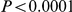; Figs. 3A,B central column).

The simulated protocol included 7 probe trials (*Day 15*) during which the block A was removed whereas a block at location B was added. This manipulation aimed at testing the “insight” working hypothesis: after a first run through the shortest path P1 and after having encountered the unexpected block B, will animals try P2 (wrong behavior) or will they go directly through P3 (correct behavior)? In agreement with Tolman & Honzik's findings, simulated animals behaved as predicted by the insight hypothesis, i.e. they tended to select the longer but effective P3 significantly more often than P2 (ANOVA, 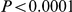; see Figs. 3A,B, right column). The patterns of path selection during this task is explained by the ability of the model to choose shortest paths. When a block is added into the environment, the goal propagation signal is also blocked at the level of the column network, and hence the simulated animals choose the shortest *unblocked* pathways.

We then tested the robustness of the above behavioral results with respect to the size of the environment. We considered a 4∶1 scaled version of Tolman & Honzik's maze and we compared the performances of 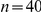 simulated animals with intact 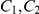 populations (“control” group) against those of 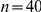 simulated animals lacking the 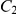 cortical population (“no 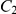
” group). The latter group did not have the multilevel encoding property provided by the 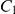
–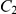 recurrent dynamics (see Sec. Recurrent cortical processing for multilevel topological mapping). Fig. 3C compares the average path selection responses of the two simulated groups across the different phases of the protocol. During *Day 1* (i.e. no blocks in the maze) both groups selected the shortest path P1 significantly more often (ANOVA, 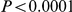; Fig. 3C left). However, the action selection policy of subjects without 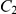 began to suffer from mistakes due to the enlarged environment, as suggested by lower median value corresponding to P1. During *Days 2–14* (with block A), the group without 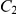 did not succeed in solving the *detour* task, because no significant preference was observed between P2 (shortest pathway) and P3 (ANOVA, 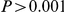; Fig. 3C center). By contrast, control animals coped with the larger environmental size successfully (i.e. P2 was selected significantly more often than P3, ANOVA, 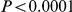). During the probe trials of *Day 15* (with a block at B but not at A), the group without 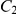 was impaired in discriminating between P2 and P3 (ANOVA, 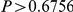; Fig. 3C right), whereas control subjects behaved accordingly to the insight hypothesis (i.e. they selected the longer but effective P3 significantly more than P2; ANOVA, 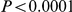). The better performances of control subjects were due to the fact that back-propagating the goal signal through the cortical network benefited from the higher-level representation encoded by the 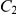 population and from the 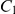-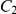 interaction during planning (see Supplementary Text S1 Sec. Exploiting the topological representation for navigation planning, Fig. S2). Indeed, an intact 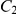 population allowed the goal signal to decay with a slower rate compared to 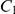, due to the smaller number of intermediate columns in 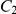 (i.e. planning could benefit from a more compact topological representation).

Henceforth we demonstrate how the modeled neural processes can be interpreted as elements of a functional network mediating spatial learning and decision making. We show that the neural activity patterns of all types of neurons in the cortical model are biologically plausible in the light of PFC electrophysiological data [20], [35], [59]–[66].

### Single cell and population place codes

#### Analysis of single cell receptive fields

**Figure 4:**
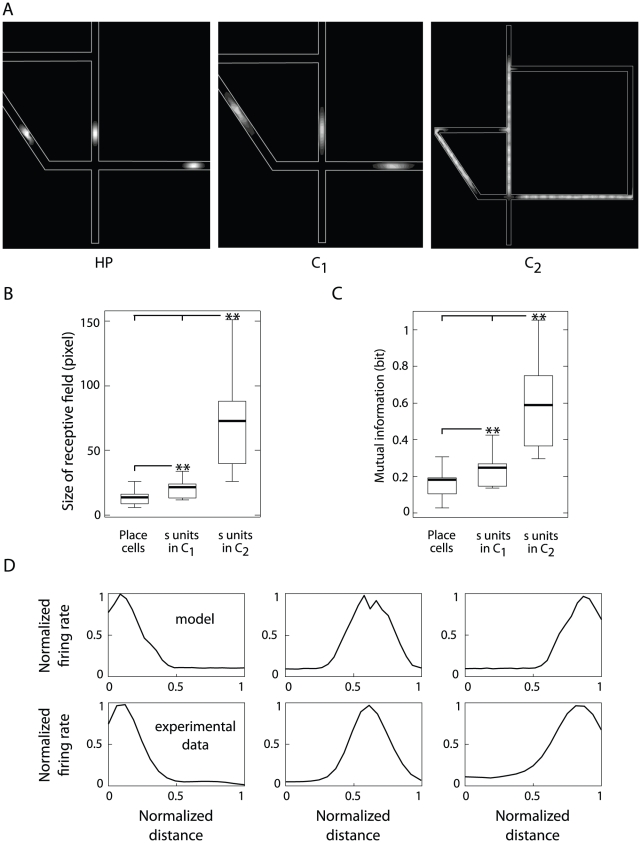
Single cell response analysis. Simulation results and relation to electrophysiological PFC recordings. (**A**) Examples of receptive fields of model hippocampal place (HP) cells (left), cortical neurons 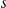 in 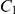 (center) and 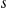 in 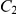 (right) when the simulated animals were solving the 4∶1 version of Tolman & Honzik's maze. White regions denote large firing rates whereas black regions correspond to silent activity. (**B**) Mean size of the receptive fields for each neural population, measured in pixels (i.e. 5×5 cm square regions). (**C**) Mutual information between single unit responses and spatial input for each population. **(D)** Location-selective responses of model single neurons 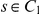 functions of the normalized distance traveled along a section of the linearized trajectory P3 (top row) and medial PFC pyramidal cells recorded from navigating rats (bottom row).

To understand how single neurons took part to place coding, we compared the location-selective activities of two types of units of the model: hippocampal place (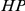) cells and cortical neurons 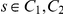 (Fig. 1). We analyzed their discharge patterns while simulated animals were solving the 4∶1 version of the Tolman & Honzik's task. Fig. 4A displays some samples of receptive fields recorded from each of these populations. The representation encoded by units 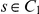 was in register with the place field organization of 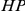 cells (left and center panels), whereas the activity of neurons 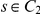 (right panel) captured some structural properties of the environment (i.e. alley organization). As quantified on Fig. 4B, the mean size of place fields increased significantly as spatial information was subsequently processed by 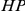, 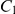 and 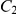 populations (ANOVA, 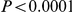; see also Fig. S3 A for results based on a kurtosis analysis, Supplementary Text S2). These findings are consistent to experimental data on the sizes of receptive fields of hippocampal and PFC cells recorded from rats solving a navigational task [20].

We also characterized the multistage spatial processing of the model in terms of Shannon mutual information between single unit responses and spatial locations (Supplementary Text S2). As shown on Fig. 4C, the activity of neurons 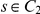 encoded, on average, the largest amount of spatial information, followed by neurons 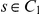 and 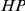 cells (ANOVA, 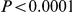). This relationship was due the fact that the smaller the receptive field is, the larger is the region of the input space for which a neuron remained silent, and then the lesser can be inferred about the entire input set by observing the variability of the neuron discharge. This result was based on the computation of the total amount of information, averaged over all positions. Other authors characterized the spatial locations where cells are most informative, such as the spatial coherence, which estimates the local smoothness of receptive fields [20], or the local information, which is a well-behaved measure of a location-specific information [67], [68].

We also compared the location-selective responses of single neurons 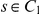 with the discharge patterns of pyramidal cells recorded from the medial PFC of navigating rats (see Materials and Methods Sec. Statistical analysis of neural activities). Fig. 4D shows three examples of experimental (top) and simulated (bottom) receptive fields evenly distributed on a linear alley. Real and simulated patterns are consistent to each other in terms of both shape and signal-to-noise ratio of the response profiles. These results corroborated the hypothesis that purely location-selective neurons 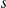 of the model might find their biological counterpart in real PFC populations.

#### Analysis of population place coding properties

**Figure 5:**
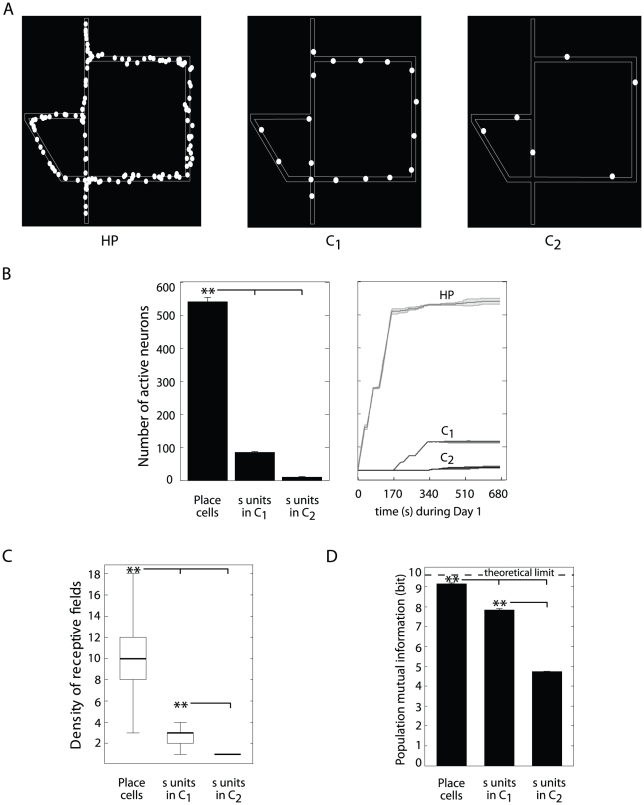
Population place coding analysis. Simulation results. (**A**) Examples of distributions of place field centroids for the populations of model HP cells (left), cortical neurons 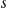 in 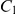 (center) and 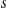 in 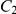 (right), when simulated rats were solving the 1∶1 version of Tolman & Honzik's maze. (**B**) Mean number of active neurones (average over 40 animals) when learning the 4∶1 Tolman & Honzik's maze (left). Evolution of the number of active neurons during the first 12 trials, i.e. Day 1 (right). (**C**) Mean spatial density (averaged over 40 animals) of receptive fields for each neural population. **(D)** Mutual information between population responses and spatial input states.

As aforementioned, we modeled the interplay between hippocampus and PFC to produce compact space codes suitable to support navigation planning. Fig. 5 shows how the implemented multistage processing (including the 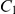
–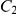 recurrent dynamics) provided a progressive sparsification of the population place code. Fig. 5A qualitatively compares three examples of distributions of receptive field centers of 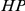 and 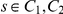 neural populations (left, center and right, respectively). Consistently to experimental findings reported by Jung et al. [35], our simulated cortical units produced less redundant place representations than 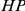 cells. The size of neural populations encoding the Tolman & Honzik's maze decreased significantly from 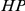 to 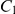 and then to 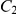 (ANOVA, 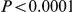; Fig. 5B). The sparser nature of cortical place codes was confirmed by the significant difference between spatial densities of receptive fields (Fig. 5C; see also Figs. S3 B,C for the results of population kurtosis and information sparseness analyses, respectively).

Finally, we measured the Shannon mutual information between population response patterns and spatial locations. The highly redundant 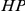 code had the largest spatial information content (ANOVA, 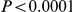; Fig. 5D). Yet, although less redundant, the population of neurons 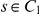 encoded about 85% of the theoretical upper bound, which proved to be suitable for solving the behavioral tasks. A significant loss of information content was observed for the population code implemented by neurons 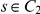. This is consistent with the functional role of the 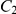 cortical network, which could not support navigation planning alone, but rather complemented the 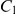 representation by encoding higher level features of the environment.

### Time course analysis of neural responses supporting decision making

#### Goal distance coding

**Figure 6:**
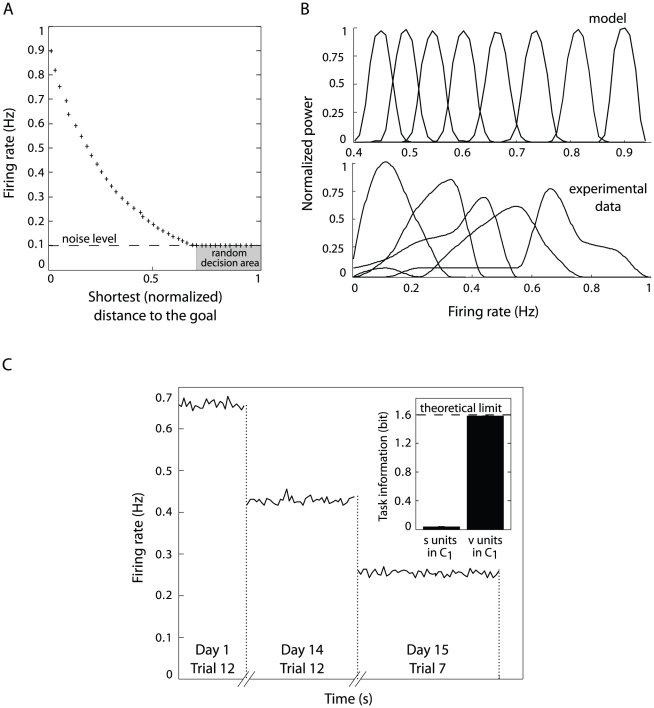
Coding of distance-to-goal and task-related information. Simulation results and relation to experimental PFC recordings. (**A**) Relation between the shortest distance of a place to the goal and the firing rate of the neuron 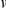 in 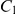 belonging to the column representing that location. Each cross corresponds to one neuron 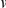. Beyond a certain distance, the intensity of the back-propagated goal signal reaches the noise level. As a consequence, neurons 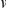 discharges become uncorrelated with the distance to the goal, and random decisions are made. (**B**) Frequency-selective responses of model single neurons 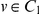 (top row) and of medial PFC pyramidal cells recorded from navigating rats (bottom row). (**C**) Relation between task-related information (Day 1 Trial 12: end of “no block” phase, Day 14 Trial 12: end of “block A” phase and Day 15 Trial 7: end of “block B” phase) and firing rate of the neuron 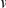 in 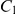 belonging to the column representing the first intersection point. Inset: mutual information between the phase of the task and single unit responses of 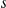 in 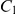 vs. 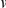 in 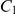.

Besides the spatial correlates of 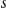 neurons' activity, the model cortical representation encoded reward-dependent information. Fig. 6A shows the correlation between the firing rate of units 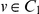 and the shortest distance-to-goal. The diagram shows that, given a location in the maze, the smaller the length of the shortest goal-directed pathway was, the larger was the mean discharge of the 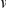 neuron belonging to the column corresponding to that location. This property was relevant to the decision making process determining the spatial navigation behavior reported in Sec. Spatial behavior in Tolman & Honzik's detour task. When the exponentially decaying frequency of 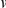 units reached the basal neural noise level, the action selection policy reduced to random search (see the performance of “*no* 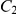
” simulated animals on Fig. 3C, central and right panels). The distance-to-goal coding property of 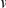 neurons called upon their selective responses in the frequency domain. The population spectral power of Fig. 6B (top) demonstrates that each neuron 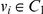 had a unique preferred discharge frequency 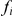 correlated to its distance-to-goal (Fig. 6A). Preferred frequencies 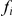 were uniformly distributed over the normalized range 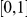. Interestingly, when we analyzed the activity of PFC pyramidal cells recorded from navigating rats (see Sec. Statistical analysis of neural activities) we found a subset of neurons with no spatial correlate but with evenly distributed preferred discharge frequencies (see Fig. 6B, bottom, for few examples). To summarize, in contrast to location-selective neurons 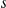 of the model, the activity of neurons 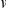 had characteristic discharge frequencies and encoded distance-to-reward information. During planning (i.e. the “mental” evaluation of multiple navigation trajectories), this property of 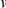 neurons allowed the value of each state to be assessed with respect to its relevance to goal-oriented behavior, consistently with PFC recordings showing reward-dependent activity patterns [61], [63].

Fig. 6C shows how the activity of neuron 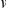 belonging to the column associated to the first intersection of Tolman's maze changed according to the task (phase of the protocol). Recall that the activity of neuron 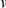 was anti-correlated to the shortest distance to the goal among available pathways (Fig. 6A). Thus, when at the end of Day 1 (i.e. Trial 12) the system learned to select the shortest path P1 (no block was present in the maze), neuron 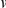 exhibited the largest firing rate. When path P1 was blocked (e.g. Day 14 Trial 12), the length of the shortest available pathway (i.e. P2) increased, as indicated by the lower discharge rate of 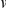. Finally, the distance to the goal was the largest when both P1 and P2 were blocked (e.g. Day 15 Trial 7). Consequently, the weakest activity of 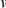 corresponded to the available path P3. In order to quantify this coding property, we measured the mutual information 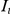 between the phases of the task and the discharge patterns of neurons 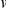 (we took neurons 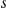 as a control population). As shown in the inset of Fig. 6C, 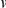 neurons (unlike 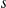 neurons) provided a significant account of abstract task-related information, meaning that the phase of the protocol could be decoded reliably by observing the time course of their discharge patterns.

#### Coding of action-reward contingency changes

**Figure 7:**
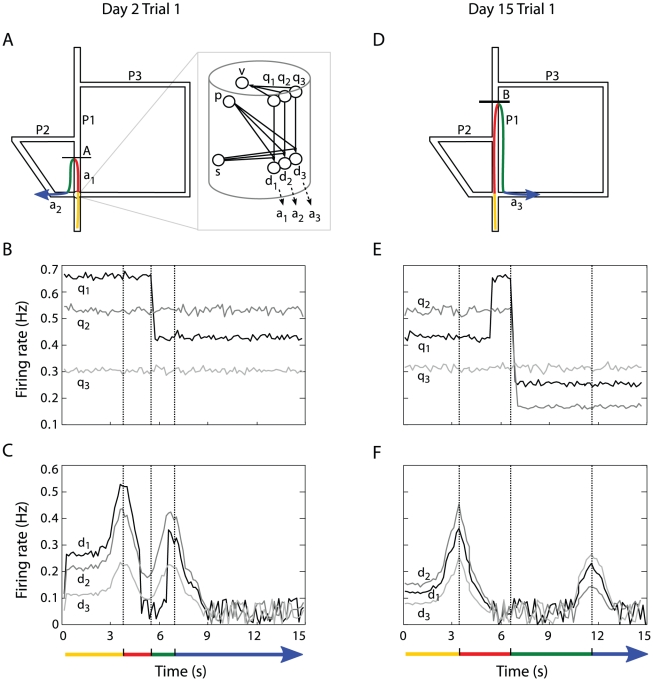
Time course analysis of action-reward contingency changes. Simulation results. Left column: Day 2 Trial 1 with block at A. Right column: Day 15 Trial 1 with block at B. **(A, D)** Examples of trajectories performed by simulated animals when encountering either block A or block B (distinct colors illustrate distinct actions). **(B, E)** Time course profile of firing rates of three neurons 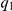, 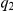 and 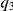 belonging to the column encoding the first intersection (and, in particular, to the minicolumns representing the actions 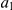, 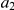 and 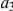, respectively). Vertical dotted lines indicate decision-making events (according to colored arrows at the bottom). **(C, F)** Time course profile of neural activity of three neurons 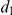, 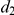 and 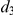 belonging to the column representing the first intersection and to the minicolumns representing the actions 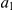, 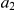 and 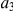, respectively.

We studied how the activity of neurons 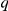 and 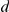 of the model contributed to decision-making. Recall that, after learning, each cortical minicolumn 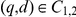 encoded a specific state-action pair 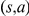. The analysis reported on Fig. 7 shows the time course of the firing rate of units 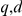 belonging to the column coding for the first intersection of Tolman & Honzik's maze. Figs. 7A,B,C focus on the action selection process taking place at the beginning of Day 2 Trial 1 of training (i.e. with block A). During the outward journey, the simulated animal arrived at the intersection point at 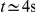. Due to the policy learned during Day 1 of training (i.e. without any block in the maze), at 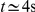 the unit 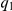 of the minicolumn associated to the action leading to P1 discharged with the largest firing rate, followed by unit 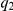 of the minicolumn associated to P2, and finally by 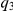 related to P3 (Fig. 7B). Thus, corresponding neurons 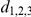, which combined inputs from 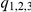, respectively, with the location-selective activities of neurons 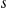 of the same column, discharged according to the same ranking at 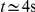 (Fig. 7C). As a consequence, the action driven by 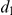 was selected and the simulated animal proceeded along P1. However, when block A was encountered at 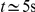, the model updated the topological representation (see Supplementary Text S1 Sec. Spatial learning: encoding topological representations), which resulted in a change of action-reward contingencies (with 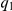 firing rate dropping below that of 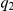, meaning that the action leading to P2 from the intersection point was now better scored, Fig. 7B). This activity update is consistent with findings showing sustained discharge changes highly sensitive to a switch in reward contingencies [37], [66]. Thus, when during the backward journey the animal met again the intersection point (at 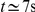), neuron 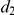 discharged with the largest frequency (Fig. 7A, bottom) leading to the selection of P2.

Similarly, the analysis reported on Figs. 7D,E,F shows how the time course of the relative strengths of the activities of neurons 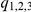 and 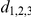 determined action selection at the beginning of the probe test, Day 15 Trial 1 (with block A removed and block B inserted). Notice the increased 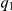 firing frequency at 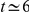 s reflecting the re-discovery of the transition blocked at A during Days 2–14 of training.

#### Coding of prospective place sequences

**Figure 8:**
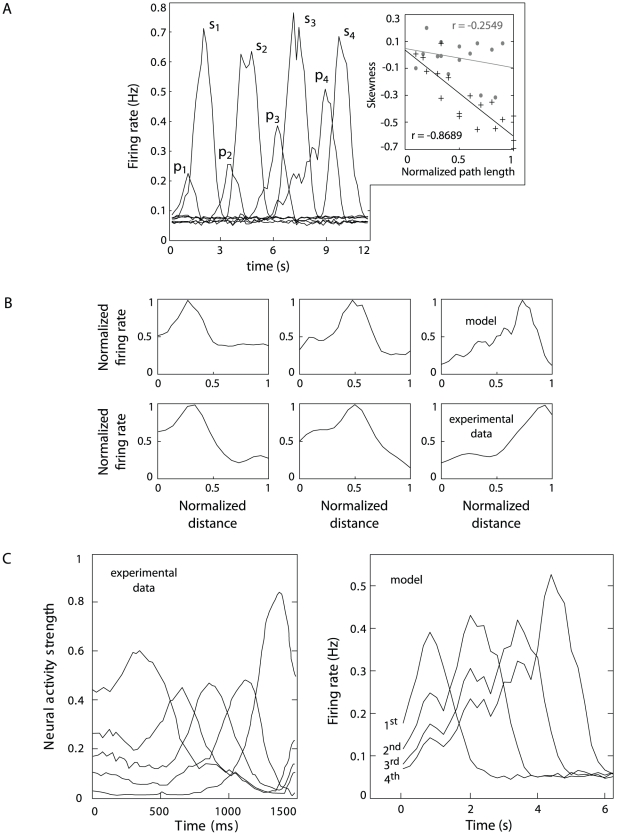
Coding of prospective place sequences. Simulation results and relation to experimental PFC recordings. (**A**) Comparison of time course shapes of the responses of four pairs of neurons 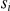 and 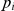 belonging to the same column (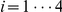). Inset: correlation between the position of a given column within a planned path (measured as the path length from the starting column to that given column) and the skewness of the time course profile of its neuron p activity (black crosses) or its neuron s activity (gray dots). (**B**) Asymmetric responses of model single neurons 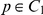 (top row) and of pyramidal cells recorded from the PFC of navigating rats (bottom row). (**C**) Sequence order coding carried out by a population of monkey PFC neurons (left; data courtesy of Averbeck et al. [65]). Each curve denotes the strength of the neural activity encoding a specific segment of a planned drawing sequence (the peak of each curve corresponds to the time when the segment is actually being drawn). Similarly, a sequence order coding property was observed when recording neurons 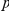 in 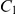 of the model (right). Each curve measures the activity of a neuron 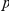 belonging to a planned trajectory. The peaks of activity represent the times when places are actually visited.

After a local decision was made (based on the competition between 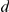 neurons' discharges), collateral projections 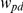 (Fig. 1B and Fig. S2 A) enabled the cortical network to forward propagate the selected state-action sequence. Fig. 8 shows how the time course of 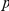 neurons' firing patterns subserved this propagation process. First, we analyzed the receptive fields of 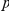 units as the simulated animal proceeded from the starting position towards the goal. Fig. 8A compares the activity profiles of neurons 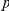 and 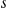 belonging to the same columns (four different columns are considered in this example). In contrast to the symmetrical receptive fields of neurons 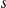 (see also Fig. 4), all neurons 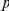 had asymmetric response profiles with negative skews (i.e. with the left tail of the distribution longer than the right tail). The skewness of these neural responses increased quasi-linearly with the number of synaptic relays forming a mentally planned trajectory (Fig. 8A, top-right inset). When we analyzed PFC data recordings from navigating rats (see Materials and Methods, Sec. Statistical analysis of neural activities), we also found a subset of neurons with asymmetric tuning curves, whose negative skewness seemed to be correlated to the distance traveled by the animal (Fig. 8B).

Another difference between neurons 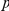 and 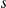 of the model was that the peak discharge frequency of neurons 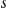 did not have any significant modulation, whereas all neurons 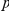 had mean peak firing rates positively correlated to the distance traveled towards the goal (Fig. 8A). Accordingly, Jung et al. [35] provided experimental evidence for increased neuronal firing rates during the approach to a reward. Finally, an important property of neurons 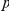 of the model is that their discharge tended to temporally anticipate the activity of neurons 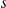 (Fig. 8A). In other words, 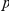 neurons encoded prospective place information predicting the next state visited by the animal. A cross-correlogram analysis showed that 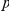 neurons' activity anticipated the discharge of 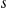 neurons by a mean time delay 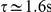, 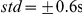 (given a constant velocity of 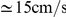). The prospective coding property of neurons 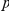 is consistent with experimental findings on PFC recordings reported by Rainer et al. [62].

We further studied the predicting nature of 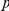 neurons' activity in relationship to experimental data on neural encoding of the serial order of planned sequences before the action begins [65]. In their experiment, Averbeck et al. [65] performed simultaneous recordings of PFC single cell activities from monkeys drawing sequences of lines (i.e. segments forming abstract shapes). Each segment was associated to a distinct pattern of neural activity, and the relative strength of these patterns *prior* the actual drawing was shown to predict the serial order of the sequence of segments actually drawn by monkeys (Fig. 8C left). Consistently, we found that the ranking of the discharge frequencies of 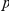 neurons *before* the actual execution of a planned trajectory was a good predictor of the serial order of the states to be visited by the simulated animal (Fig. 8C right). This relationship not only held at time 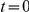 (i.e. at the very beginning of a trajectory), but for every time 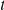, meaning that the ranking of 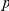 neurons' firing rates could predict the order of future state sequences at any moment.

### Comparative analysis of model and experimental PFC population activity patterns

We studied to what extent the neural populations of the model (i.e. 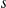, 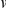, 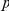, 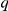 and 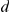 neurons) could be quantitatively segregated on the basis of a set of statistical measures. We then compared the results to those obtained by applying the same clustering analysis to a population of neurons recorded from the medial PFC of navigating rats (see Materials and Methods Sec. Statistical analysis of neural activities).

**Figure 9:**
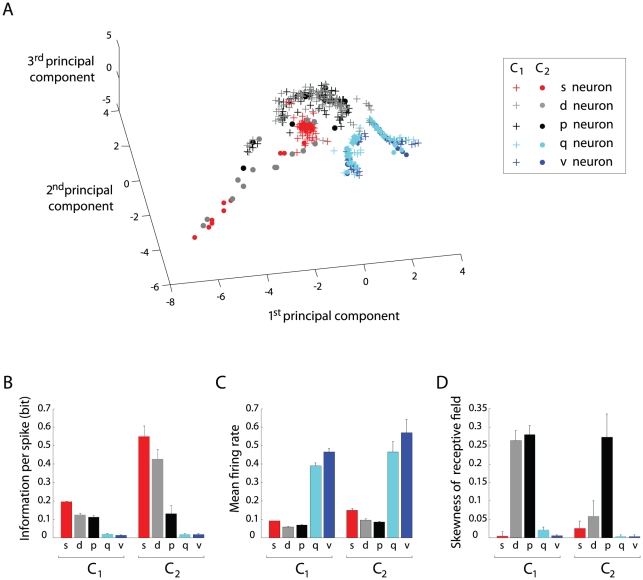
Principal component analysis of simulated neuronal activities. (**A**) Simulated neurons represented within the space defined by the first three principal components. (**B**) Spatial information per spike averaged over each neural population of the model. (**C**) Mean firing rate averaged over each neural population. **(D)** Mean absolute skewness average over each population. The color code is the same used in (A).

We first gathered all non-silent simulated neurons recorded during the 4∶1 version of Tolman & Honzik's task. All types of units (i.e. 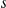, 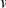, 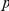, 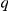, 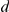) were pulled together in a data set. We characterized each neuron's discharge by measuring its mean firing rate, standard deviation, skewness, lifetime kurtosis, spatial information per spike and spatial mutual information (see Supplementary Text S2). Then, we performed a principal component analysis (PCA) on the multidimensional space containing the values provided by these measures per each neuron (see Figs. S4 A,C for details). Fig. 9A shows the resulting data distribution in the space defined by the first three principal components. Interestingly, model neurons with different functional roles tended to occupy distinct regions of the PCA space. For instance, neurons 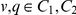, whose function in the model is to propagate goal information and code for the distance to the goal, were located within the same portion of the PCA space (blue and cyan crosses and circles). All neurons 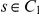, which primarily code for spatial locations, were also clustered within the PCA space (red crosses). Neurons 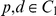 (and also 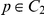), responsible for forward signal propagation and local decision making, respectively, were aggregated within the same region (gray and black crosses, and black circles). Finally, neurons 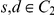, mainly involved in high-level mapping and navigation planning, were also separated from other units in PCA space (gray and red circles).

Figs. 9B,C,D display the mean values, averaged over each population 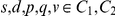 of the model, of three statistical measures (out of six) used to perform the PCA. These diagrams can help understanding the data point distribution of Fig. 9A. When considering the mean spatial information per spike (Fig. 9B), at least three groups could be observed: neurons whose activity had nearly no spatial correlate (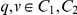), neurons conveying intermediate amounts of spatial information (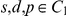 and 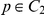), and neurons with larger spatial information values (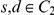). The mean firing rate parameter (Fig. 9C) allowed two distinct groups to be clearly identified: one with low average firing (neurons 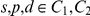), and one with high firing rates (neurons 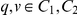). Together with Fig. 9A, this diagram can help understanding why neurons 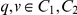, which had almost no spatial correlate and very high firing rates compared to other populations of the model, were well segregated within the same region of the PCA space (Fig. 9A, blue and cyan crosses and circles). Finally, when comparing the mean skewness values of all neural populations (Fig. 9D), neurons 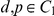 and 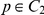 were pulled apart, according to their distribution in the PCA space (Fig. 9A, gray and black crosses, and black circles). As a control analysis, we extended the data set used for the PCA by adding a population of neurons with random Poisson activities. As shown in supplementary Figs. S5 A–B, the population of Poisson neurons (light green data points) was well separated from all model neurons within the space defined by the first three principal components, suggesting that the variability of model discharge properties could not be merely explained by a random Poisson-like process.

**Figure 10:**
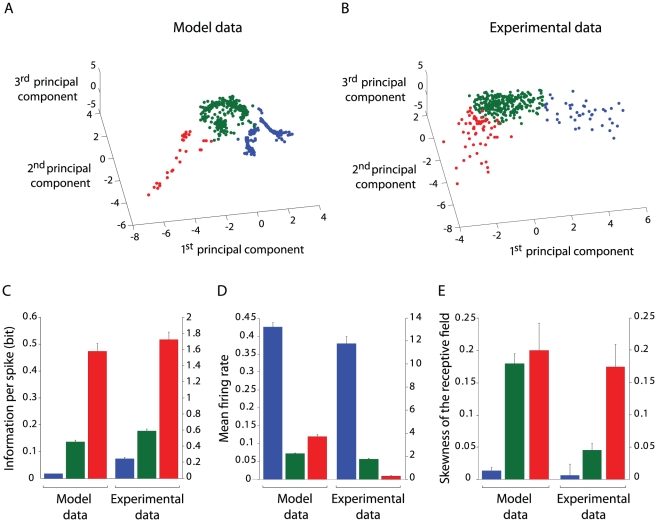
Principal component analysis and unsupervised clustering of simulated and real neuronal activities. (**A**) Clustering of model activities within the PCA space. The same color scheme (used to discriminate clusters) is applied throughout the entire figure. (**B**) Blind clustering of real PFC recordings represented in the three first principal components space. **(C, D, E)** Mean information per spike, firing rate and skewness for real vs. model subpopulations (i.e. clusters).

We then applied an unsupervised clustering algorithm (k-means clustering method with 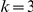) to partition the distribution of data points of Fig. 9A, based on the discharge characteristics of model neurons. This blind clustering analysis (i.e. without any a priori knowledge on neural populations) allowed us to identify three main groups (Fig. 10A). The first cluster (blue data points) corresponded to non-spatial, reward-related neuronal activities (i.e. neurons 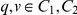). The second cluster (green points) represented location-selective activity (mainly from neurons 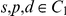, but also including some neurons 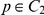). The third cluster (red data points) corresponded to location-selective activity of neurons in the cortical network 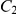 (i.e. mainly 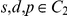). See supplementary Fig. S6 for details on the composition of the three identified clusters.

We performed the same series of analyses on a dataset of medial PFC neurons recorded from navigating rats (see Materials and Methods, Sec. Statistical analysis of neural activities). We characterized every recorded activity according to the same set of statistical measures used for model neurons (i.e. mean firing rate, standard deviation, skewness, lifetime kurtosis, spatial information per spike and spatial mutual information, see Supplementary Text S2). Then, we applied a PCA on the resulting high dimensional space containing, per each neuron, the resulting values of these measures (see Figs. S4 B,D for details). Finally, we used the same unsupervised k-mean clustering algorithm to partition the data distribution in the space defined by the first three principal components. As for simulated data, the clustering method identified three main classes (Fig. 10B; with red, green, and blue data points corresponding to three subsets of electrophysiologically recorded activities in the PFC). We then compared model and experimental clusters (Figs. 10C,D,E) in order to investigate whether real and simulated data points belonging to the same clusters shared some discharge characteristics. In terms of mean spatial information (Fig. 10C), we found similar non-homogeneous distributions between model and real clusters. Both red clusters encoded the largest spatial information content. Recall that the model red cluster mainly contained activities from location-selective neurons 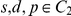 (as quantified in supplementary Fig. S6 B). When looking at mean firing rates averaged over each cluster (Fig. 10D), we found that both real and simulated activities within the blue clusters had significantly larger frequencies than others. The model blue cluster was mainly composed by neurons 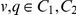 propagating reward-related information. Finally, when comparing the mean absolute values of the skewness of receptive fields (Fig. 10E), we found both model and experimental populations with asymmetric fields (i.e. non-zero skewness). Model-wise, the red and green clusters (containing neurons 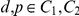, Fig. S6 B) had the largest mean skewness. Similarly, experimental red and green subpopulations had larger skewness values than the blue population. As a control analysis, we computed the three mentioned measures (i.e. information per spike, mean firing rate and skewness of the receptive field) for two populations of neurons with random Uniform and Poisson activities. As shown in supplementary Fig. S7, unlike model data, the two populations of random neurons could not explain the experimental data in terms of information content and skewness of the receptive field. Taken together, these results indicated that, within the data set of experimental PFC recordings, subpopulations of neurons existed with distinct discharge properties, and that these subpopulations might be related to distinct functional groups predicted by the model.

## Discussion

We presented a model focusing on navigation planning mediated by a population of prefrontal cortical columns. During exploration of a new environment, the model learns a topological representation in which each place is encoded by a neocortical column and strengthening of synapses between columns is used to represent topological links between places. During goal-oriented trajectory planning, an activation diffusion mechanism produces a spread of activity in the column population leading to selection of the shortest path to the goal. Our simulation results demonstrate that the model can reproduce rodent behavior previously attributed to the animals' ability to experience a cognitive “insight” about the structure of the environment [4]. Moreover, we show that spatial planning in our model is invariant with respect to the size of the maze. This property relies on the ability of the model to encode cognitive maps with a resolution that fits the structure of the environment (e.g. alleys).

On the neural level, we characterized the activities of model neurons and compared them to electrophysiological data from real PFC neurons. Our neural response analysis suggests how the interplay between the model hippocampus and the prefrontal cortex can yield to the encoding of manifold information pertinent to the spatial planning function, including, for example, distance-to-goal correlates. The model also provides a functional framework for interpreting the activity of prefrontal units observed during performance of spatial memory tasks [20], [35], [60]–[63], [65], [66]. In general, our results are consistent with the hypothesis that cognitive control stems from the active maintenance of patterns of activity in the PFC that represent goals and means to achieve them [64].

### Related work

Our model is based upon three main assumptions. First, the model relies on the columnar organization of the cortex. Although this concept is supported by many experimental studies [45],[48], no clear general function for columns has emerged to explain their role in cortical processing [69]. In addition, Rakic [70] stressed that the size, cell composition, synaptic organization, expression of signaling molecules, and function of various types of columns are dramatically different across the cortex, so that the general concept of column should be employed carefully. In our model, we call “column” a local micro-circuit composed by neurons processing common spatial information, and we propose that this columnar organization may be a substrate suitable to implement a topological representation of the environment. Second, our planning model relies on an activation diffusion mechanism. At the neural level, this suggests that strong propagation of action potentials should occur in the neocortex. This is not a strong assumption, since several studies have demonstrated such phenomena as propagating waves of activity in the brain [71], [72]. For example, Rubino et al. [73] suggested that oscillations propagate as waves across the surface of the motor cortex, carrying relevant information during movement preparation and execution. Third, the multiscale representation is based on a putative 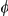 signal. There are several potential candidates for its implementation in the brain. One of these candidates is habit learning involving the striatum [74], [75]. Indeed, if for instance the rat always turns left at a particular location it may acquire a corresponding habit. The neural activity corresponding to this stimulus-response association may serve as the 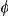 signal. In this case, the time scale of learning in the 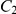 population should correspond to the time scale of habit acquisition (potentially many trials, see e.g. [75]).

Topological map learning and path planning have been extensively studied in biomimetic models (see [6] for a general review; see also [76] for theoretical discussions on hierarchical cognitive maps). These models aimed at either providing more efficient path planning algorithms or, like our work, establishing relations between anatomical substrates, electrophysiology and behavior. In particular, several studies took inspiration from the anatomical organization of the cortex and used the activation diffusion mechanism to implement planning. Burnod [49] proposed one of the first models of the cortical column architecture, called “cortical automaton”. He also described a “call tree” process that can be seen as a neuromimetic implementation of the activation diffusion principle. Some subsequent studies employed the cortical automaton concept [77], [78], while others used either connectionist architectures [16], [79]–[84] or Markov decision processes [85]. Our approach is similar to that of Hasselmo [44], who also addressed goal-directed behavior by modeling the PFC columnar structure. Both Hasselmo's and our model architectures employ minicolumns as basic computational units to represent locations and actions, to propagate reward-dependent signals, and mediate decision making. Yet, the two models differ in the encoding principles underlying the learned representations, which generate, consequently, distinct behavioral responses. The connectivity layout of Hasselmo's model allows state-response-state chains to be encoded, with each minicolumn representing either a state or an action. In our model, a state and its related actions are jointly encoded by a set of minicolumns within a column. Similar to Koene and Hasselmo [44], [86], we compared the discharge of simulated PFC units against experimental recordings exhibiting place-, action- and reward-related correlates. As explained henceforth, we focused further on the functional relationship between the hippocampus and the PFC in encoding complementary aspects of spatial memory, with a quantitative approach based on the analysis of statistical properties and information content of the neural codes. We also put the emphasis on the time course analysis of neural responses mediating place coding *vs.* decision making.

### Differential roles of PFC and hippocampus in spatial learning

The successful performance of our model in large environments relies on its ability to build a multiscale environment representation. This is in line with the proposal by McNamara et al. [87] who have suggested that humans can solve complex spatial problems by building a hierarchical cognitive map, including multiple representations of the same environment at different spatial scales. Moreover, animals may be able to chunk available information and build hierarchical representations to facilitate learning [88]–[92]. Recently, multiscale spatial representations have been identified at the neural level. For example in the entorhinal cortex, Hafting et al. [13] have shown that grid cells have spatial fields forming grids with different spacing and place field sizes. Kjelstrup et al. [93] have provided neural recordings of place cell activities in a large maze, supporting the multiscale coding property in the hippocampus. These entorhinal and hippocampal multiscale representations are likely to encode spatial contextual information at variable resolution. Complementing this code, we suggest that multiscale representations related with space, action and reward should also be found in neocortical areas such as the prefrontal cortex, commonly associated with high-level cognitive processes. Moreover, there are several works suggesting a role of the PFC in the learning of hierarchical representations. For example, Botvinick [94] reviewed how the hierarchical reinforcement learning framework [95] could explain the mechanism by which the PFC aggregates actions into reusable subroutines or skills. The multiscale property is applied there for actions instead of states as in our approach. From a biological point of view, recent studies directly pointed out the role of the PFC for hierarchical representations, with a possible anatomical mapping of the hierarchical levels along the rostro-caudal axis of the PFC [96].

In spite of a possible complementary role for the PFC and the hippocampus in multiscale space coding, our work focuses on different roles of the PFC and the hippocampus in the planning process. Namely, we propose that the hippocampus is more involved in the representation of location [1] and, possibly, route learning [97], [98], while the PFC is responsible for topological representations and action selection. From a more general perspective, a route could be seen as an example of navigation from a point to another, an episode. In contrast, the more integrated topological representation would be more similar to a set of navigation rules. This hypothesis is in accordance with data showing that the hippocampus would be involved in instance-based episodic memory, whereas the PFC would be linked to rule learning from examples [99]–[101].

Our model is consistent with recent studies suggesting a role for the PFC in prospective memory [102], [103]. Goto and Grace [102] showed that, depending on the dopamine receptors activation, PFC either incorporates retrospective information processed by the hippocampus or processes its own information to effect preparation of future actions. This is in accordance with our model which includes hippocampal information to localize itself in the environment, and then propagates reward signal to generate a goal-directed sequence of action. Moreover, Mushiake et al. [104] showed that activity in the PFC reflects multiple steps of future events in action plans. They suggested that animals may be engaged in planning sequences in a retrograde order (starting from the goal to the first motion), in conjunction with a sequence planning with an anterograde order. At the cognitive level, the activation diffusion planning process provides a capacity of mental simulation of action selection: the back-propagated goal signal allows possible navigation trajectories to be identified, whereas the forward-propagated path signal actually simulates the execution of the selected action sequence. Schacter et al. [103] recently reviewed theories on simulation of future events and neural structures associated with this cognitive ability. They showed that the same core network, which plays a role in remembering, is also implied in mental simulation. This network involves prefrontal as well as medial temporal regions including the hippocampus, which is also involved in prospective and retrospective memory coding ([105], [106]; see also [107], [108] for theoretical works modeling the role of this core network in memory retrieval and mental imagery).

### From neural activity in the PFC to behavior

Our simulation results on the Tolman and Honzik detour task show that the behavior of the model is consistent with an “insight” demonstrated by rats in this task. The insight, as defined by Tolman and Honzik, is the ability to conceive that two paths have a common section, and so when a passage through the common section is blocked, both of these paths are necessarily blocked and a third, alternative pathway, should be chosen. The realization that a common section exists leads to two conclusions. First, animals do not act exclusively according to stimulus-response associations, but use some kind of mental representation of the environment [109]. For example, in the conditions of the detour task (Fig. 2A), the rats chose path 3 without actually testing path 2 during probe trials and so they did not have a chance to form the correct stimulus-response associations to solve the task. In order to choose the correct path 3, rats had to mentally replay path 2 and realize that it was blocked, suggesting the existence of a spatial representation. Second, a representation of the environment in terms of *routes* is not sufficient to solve the task. Indeed, if after training animals store separate representations of routes via paths 1–3, then the fact that route 1 is blocked should not lead to the conclusion that route 2 is also blocked. In summary, the results of this experiment suggest the existence of a *topological graph*-like representation in which common points (nodes) and common sections (edges) are identified. The model presented here proposes a plausible way of how such a representation can be built (see below). In terms of the model, the insight capability in the detour task is mediated by the propagation of the goal signal through the nodes of the spatial graph, in which the common section of paths 1 and 2 is blocked.

The other important question addressed by the present study is whether the requirements of the proposed model are consistent with the neural activities observed in the PFC. We show that *all* types of neurons that are required by the model, have actually been observed in the PFC. Namely, *(i)* the state-encoding 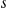 neurons in the model correspond to spatially selective prefrontal neurons with different receptive field sizes (Fig. 4D, see also [20]); *(ii)* the distance-to-goal, or value, neurons 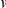 correspond to the PFC neurons with constant discharge rate (Fig. 6B), giving rise to the prediction that neurons with higher (constant) discharge rates can code for locations closed to reward; *(iii)* the prospective-coding 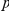 neurons in the model correspond to PFC neurons with the firing rate that increases when the animal moves toward the goal (Figs. 8B,D, see also [62], [65]); and, finally, *(iv)* neurons 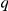 and 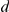, which together encode state-action values, show activity patterns similar to strategy-switching neurons observed by Rich and Shapiro [37]. Indeed, the authors reported that in their task (i.e. strategy switching in a plus-maze) during the periods before and after reward contingency change, different subsets of PFC neurons were highly active. This is exactly what was observed in our model. For example, neurons 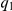 and 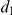 that were more active than neurons 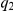 and 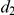 *before* the contingency change (Figs. 7B,C at 4 s) became relatively less active *after* the change (Figs. 7B,C at 5 s).

The model provided a vantage point to interpret PFC electrophysiological data in terms of quantitative clustering of population activity. On the basis of a set of statistical measures, we performed a principal component analysis on both simulated and real data sets of PFC recordings. This study gave rise to comparative results based on the identification of clusters of characteristic discharge properties. We could put forth some hypotheses about the functional meaning of the observed clusters —in terms of their role in spatial localization and planning, reward coding, and prospective memory. For instance, model neurons mediating planning in large scale mazes (i.e. belonging to the cortical population 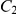 of the model) could be segregated from other simulated units (red cluster in Fig. 10). A corresponding cluster was found when analyzing real recordings, corroborating the hypothesis of the presence of neurons with similar discharge properties in the PFC. We also identified another cluster of real PFC activities which contained both pyramidal cell and interneuron responses (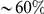 and 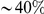, respectively). This cluster corresponded to goal propagating neurons of the model (blue cluster in Fig. 10), leading to the prediction that interneurons may contribute to decision making by participating to the propagation of information relevant to the next decision to be taken. Interestingly, in their study of spatial navigation, Benchenane et al. [60] showed that the inhibitory action of PFC interneurons onto pyramidal cells is enhanced during periods of high coherence in theta oscillations between hippocampus and PFC occurring at decision points.

### Limitations and future work

In this model, the simulated hippocampal population does not account for the full range of place cell firing properties that have been extensively studied during the past decades. Particularly, the dynamics of the model hippocampal population in terms of learning, extrafield firings and rhythmic discharges are not reproduced. Experimental data show that the introduction or the removal of a barrier in the environment may induce learning-related changes in the hippocampal population (remapping). For example, previously silent cells may discharge and previously active cells may be silent when the animal visits the modified environment [110], [111]. In addition, complementing their location selectivity, hippocampal place cells may have extrafield firings, and neural ensembles in the hippocampus may transiently encode paths forward of the animal [112]. Finally, it has been shown that hippocampal place cell discharges are modulated by theta oscillations (e.g. phase precession phenomena, [113]) and that the hippocampus and the PFC seem to synchronize at behaviorally relevant places in a maze, such as decision points [114]. Although the scope of the presented model is targeted to address the PFC firing patterns, these experimental data suggest that improving our hippocampal place cell model is relevant to provide plausible predictions about the interactions between the hippocampus and the PFC during decision making in spatial navigation tasks.

The second limitation of the model is related to the issue of goal representation in the PFC. The model makes decisions based on an appetitive motivational signal only (i.e. the reward at the goal site). Clearly, there are other variables apart from the reward size that influence the planning process. For example, there is evidence that physical efforts required to reach the goal or delay in reward delivery influence PFC-dependent behavioral decisions [115]. Moreover, the model can merely deal with a single goal at present and can not estimate relative values of different goals [63]. In order to address these limitations, the activation diffusion mechanism in the model can be extended to propagate several motivational signals, the intensities of which are proportional to their subjective values. In this case, a goal-related effort or delayed reward can be modeled by adjusting the relative values of motivation signals at different locations in the maze.

We limited our study to a structured maze (i.e. Tolman & Honzik's maze [4]) to focus on the adaptive response to dynamic blocking of goal-directed pathways, a required property to validate detour-like navigation behavior. Furthermore, Tolman & Honzik's maze provided us with the possibility to investigate the neural dynamics of the modeled network at clear decision points –i.e. at the intersections between corridors. Several models have addressed spatial navigation in open-field environments based on place-triggered-response strategies (i.e. locale navigation), in which hippocampal place cell activity is associated to the best local action leading to the goal (e.g. [57], [116], [117]). In fact, two components are relevant to avoid the combinatorial explosion of the 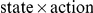 space in open-fields: *(i)* the reliability of the spatial code in terms of minimum hidden-state probability, to avoid, for instance, that a same place cell population can code for different locations –a problem often arising from sensory-aliasing phenomena in purely topological maps; *(ii)* the use of a discrete action space, meaning that a finite set of actions are available at each state (location). Our hippocampal-PFC model satisfies these requirements. We already used a highly simplified version of the model presented in this paper to solve open-field navigation problems (e.g. Morris water maze [11]). Note that, however, Dollé et al. (2010) focused on navigation strategy switching and did not model the PFC columnar organization and the (possibly) involved computational processes (e.g. multiscale coding) to drive planning behavior [11]. In open-field environments with no obstacles our model predicts 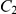-like units with uniform activity across the whole space –as a result of a uniform 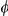 signal reflecting equal probability of turning at each location. Adding borders or barriers would result in the “recruitment” of new 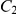 units preferentially active on either one side or the other of the barriers. In more structured environments such as interconnected arenas (e.g. [15]), the model predicts separate 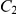 units for each space. To our knowledge, there is no direct experimental evidence in favor or against the existence of such PFC units.

Another interesting direction of future work is to study the encoding of task-related information in the PFC during sleep. Although it is likely that information is transferred during task learning, memory consolidation during sleep also appears to play a central role [59]. In particular, sharp wave-ripple complexes in the hippocampus seem prominent for transferring labile memories from the hippocampus to the neocortex for long-term storage [118]. A key issue for modeling approaches is to understand computational properties of this learning mechanism.

## Supporting Information

Figure S1Multilevel topological map learning in 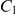 and 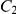 populations. Columns in 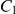 and 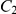 populations encode locations at different spatial resolutions. For instance, column 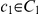 corresponds to the end of the first alley, whereas 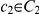 encodes the entire alley before the turn. The model achieves multilevel state coding thanks to collateral projections 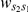 between columns in 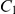 and 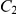. When a place transition occurs, lateral connections between columns selective for previous and next states are updated in 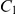 population (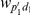 and 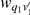), as well as in 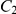 population (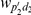 and 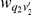). These latter synaptic weights are modified thanks to the inputs conveyed by 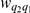 and 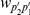 projections so that the activity of 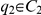 will mirror the activity of 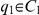, whereas 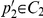 will mirror 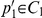. Finally, another set of collateral connections from 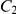 to 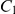 population (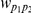 and 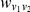) enables columns in 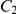 population to bias the activity in neurons 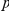 and 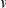 of 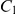 population.(PDF)

Figure S2Action planning through multilevel activation diffusion of a goal signal. (**A**) A motivation signal induces the activity of neurons 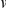 in the goal columns of 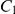 and 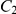 populations *(1)*. The goal information is then back-propagated through the reverse state associations encoded by neurons 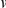 and 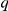 in 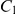 and 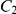 *(2)*. When the back-propagated goal signal reaches the columns selective for the current position in both 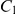 and 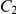 populations, the coincidence of the state-related input conveyed by 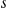 neurons and the goal-related input transmitted by 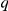 neurons activates neurons 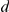 *(3)*. In turns, neurons 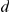 trigger the forward propagation of a pathway signal through the neurons 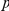 and 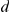 *(4)*. At each step of the forward propagation, the motor action associated to the most active neuron 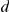 can be selected (e.g. for the first planning step 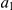 for 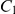 population and 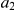 for 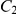 population) and the sequence of actions from the current position to the goal can be iteratively readout. (**B**) Effect of the top-down modulation exerted by the population 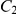 upon the back-propagating activity at the level of neurons 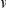 in 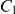. We plotted the relation between the number of synaptic relays connecting the columns that form the planned path from a given place to the goal and the firing rate of the neuron 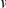 belonging to the column representing that place. Each cross indicates the activity of one neuron 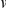 after a given number of synaptic relays. Without any modulation from the 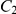 population (exponentially decreasing set of points), the activity of neurons 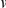 drops quickly to the noise level as the length of the planned path increases. With the 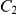 modulation, the time constant of the decreasing function is much larger, leading to a better propagation in large environments. As indicated by black rectangle, given a pathway involving 10 synaptic relays, a modulated neuron 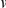 would fire at about 0.9 Hz, whereas it would only fire at about 0.35 Hz without modulation.(PDF)

Figure S3Additional measures of the location selectivity property of neurons 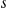 in 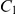 and neurons 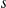 in 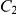. (**A**) Left: sparseness of single cell responses as measured by their lifetime kurtosis. The larger the kurtosis is, the larger is the sparseness. Right: the size of the receptive field (see Fig. 4B) is anti-correlated to the lifetime kurtosis measure. (**B**) Left: sparseness of the population place code as measured by the population kurtosis function. Right: the density of receptive fields (see Fig. 5C) is anti-correlated to the population kurtosis measure. (**C**) The spatial information sparseness –computed as the ratio between population information and the sum of single cell information– demonstrates that the hippocampal place code is redundant in terms of spatial information content. By contrast, although loosing part of the spatial information, the cortical population achieves a better coding, maximizing the contribution of each unit to the population code, particularly for the 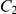 population. **(D)** Spatial information Pearson correlation. Expectedly, the way spatial information is encoded by neurons firing rates is not different between the three populations: they all have their surprise information strongly correlated with the strength of the discharge activity.(PDF)

Figure S4 Principal component analysis of simulated (left) and real (right) neuronal activities. Eigenvalues (top) and structure of the principal components (bottom).
(PDF)

Figure S5 Principal component analysis (PCA) of simulated neuronal activity. Comparison between model and random population activities. Two different views of the same three-dimensional PCA space are shown (A and B, respectively). The size of the original data set used for the analysis reported on Fig. 9 was doubled by adding a population of Poisson neurons. The distribution of the mean firing rates over the original data set was fitted by the distribution of the mean firing rates computed over the population of Poisson neurons.
(PDF)

Figure S6Principal component analysis (PCA) and unsupervised clustering of simulated neuronal activities. (**A**) Clustering of model activities within the PCA space (first three principal components). (**B**) Distribution of neural populations 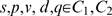 for each cluster (top: percentages; bottom: absolute counts).(PDF)

Figure S7Principal component analysis (PCA): control analysis for the comparison between experimental and model data shown in Fig. 10. (**A**) Information per spike computed for model rescaled data, experimental data and two random neuron populations (formed by 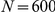 neurons each). Model data in this figure were obtained by rescaling the mean firing rates of model neurons from 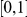 to 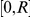, where 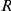 denoting the maximum mean firing rate observed in experimental data. The first random population, called “Uniform distribution”, consisted of neurons discharging between 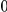 and 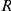 according to a uniform distribution. The second control population, called “Poisson distribution”, generated random activities following Poisson distributions with parameters (i.e. means) drawn from an uniform distribution between 0 and R. As expected, the two random populations exhibited extremely weak information content and could not explain the high spatial information found in experimental data. (**B**) Mean firing rate for the same four sets of data. This figure provides a mere empirical validation of the process used to draw random neural responses with mean firing rates within the range of those of experimental and rescaled model data. (**C**) Skewness of the receptive field for the same four sets of data. The random neural populations did not have asymmetrical deformation of their response profiles, and thus could not explain the values observed experimentally.(PDF)

Text S1 Detailed account of the model. This document provides equations and parameter settings related to the column model, the connectivity layout and the learning rules shaping the dynamics of the network.
(PDF)

Text S2 Statistical analyses of neural activities. This document provides a description of the set of statistical measures used to characterize the model neural code.
(PDF)
