# Supplementary figures and images for: Spatial Learning and Action Planning in a Prefrontal Cortical Network Model
Source: PLoS Comput Biol. 2011 May 19;7(5):e1002045. doi: 10.1371/journal.pcbi.1002045 (PMC3098199; doi:10.1371/journal.pcbi.1002045)

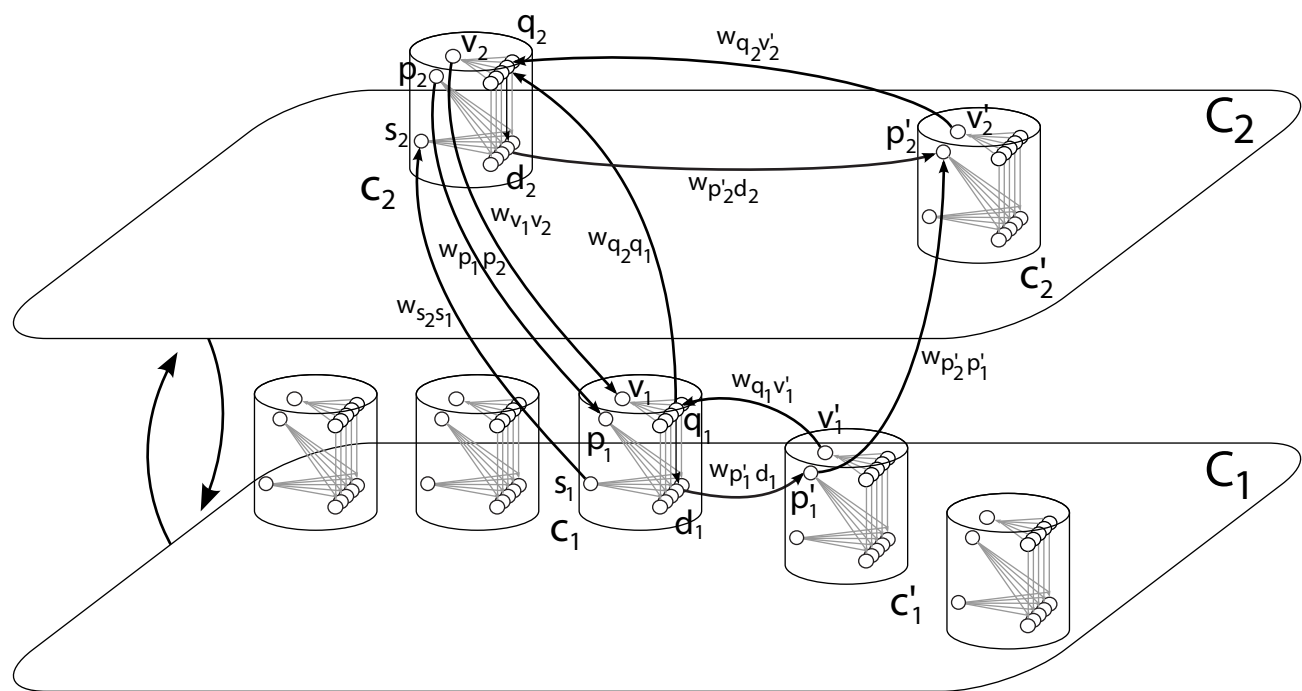

start

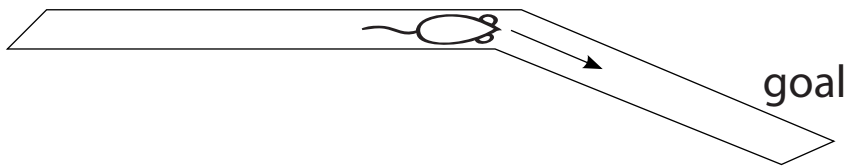

Supplement: Figure S1 — Multilevel topological map learning in and populations. Columns in and populations encode locations at different spatial resolutions. For instance, column corresponds to the end of the first alley, whereas encodes the entire alley before the turn. The model achieves multilevel state coding thanks to collateral projections between columns in and . When a place transition occurs, lateral connections between columns selective for previous and next states are updated in population ( and ), as well as in population ( and ). These latter synaptic weights are modified thanks to the inputs conveyed by and projections so that the activity of will mirror the activity of , whereas will mirror . Finally, another set of collateral connections from to population ( and ) enables columns in population to bias the activity in neurons and of population. (PDF) [file pcbi.1002045.s001.pdf]

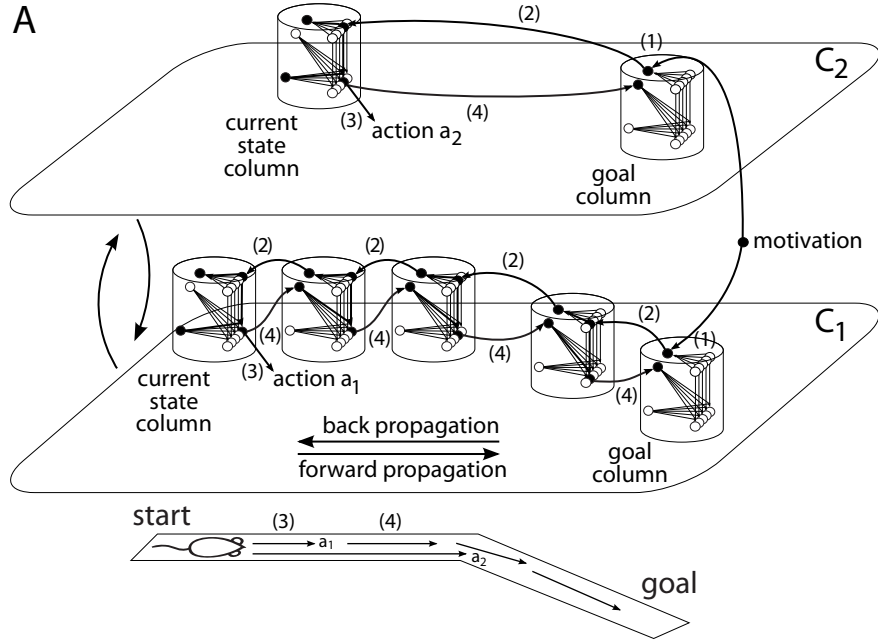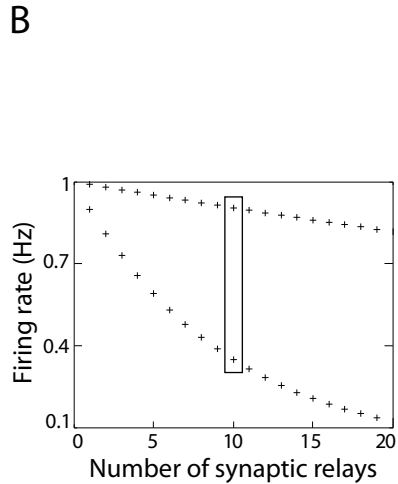

Supplement: Figure S2 — Action planning through multilevel activation diffusion of a goal signal. (A) A motivation signal induces the activity of neurons in the goal columns of and populations (1). The goal information is then back-propagated through the reverse state associations encoded by neurons and in and (2). When the back-propagated goal signal reaches the columns selective for the current position in both and populations, the coincidence of the state-related input conveyed by neurons and the goal-related input transmitted by neurons activates neurons (3). In turns, neurons trigger the forward propagation of a pathway signal through the neurons and (4). At each step of the forward propagation, the motor action associated to the most active neuron can be selected (e.g. for the first planning step for population and for population) and the sequence of actions from the current position to the goal can be iteratively readout. (B) Effect of the top-down modulation exerted by the population upon the back-propagating activity at the level of neurons in . We plotted the relation between the number of synaptic relays connecting the columns that form the planned path from a given place to the goal and the firing rate of the neuron belonging to the column representing that place. Each cross indicates the activity of one neuron after a given number of synaptic relays. Without any modulation from the population (exponentially decreasing set of points), the activity of neurons drops quickly to the noise level as the length of the planned path increases. With the modulation, the time constant of the decreasing function is much larger, leading to a better propagation in large environments. As indicated by black rectangle, given a pathway involving 10 synaptic relays, a modulated neuron would fire at about 0.9 Hz, whereas it would only fire at about 0.35 Hz without modulation. (PDF) [file pcbi.1002045.s002.pdf]

**A**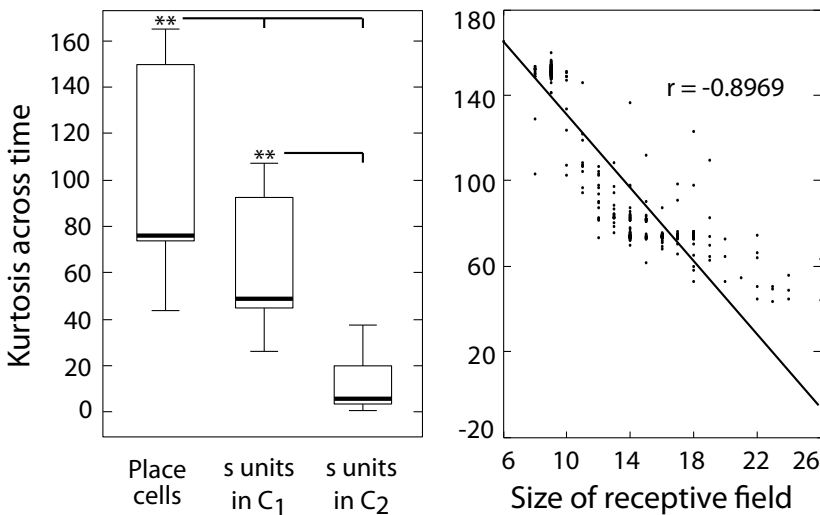**C**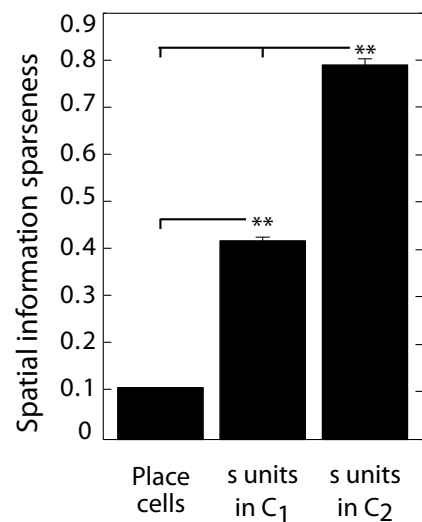**B**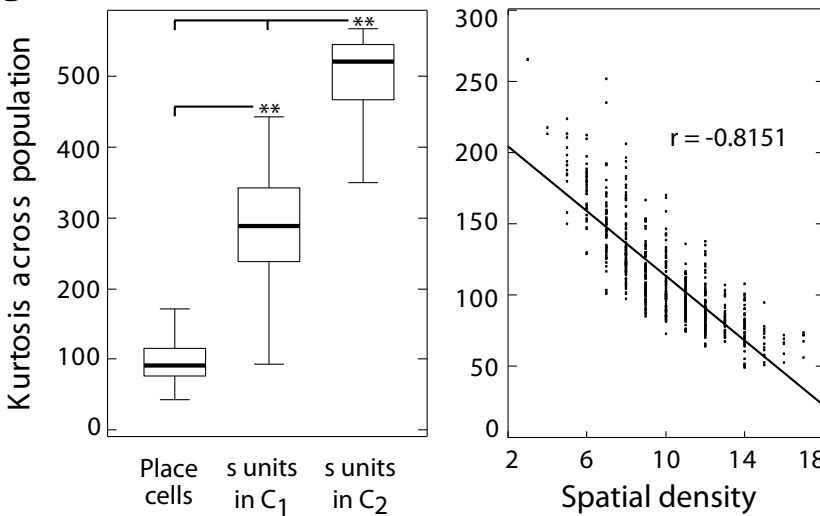**D**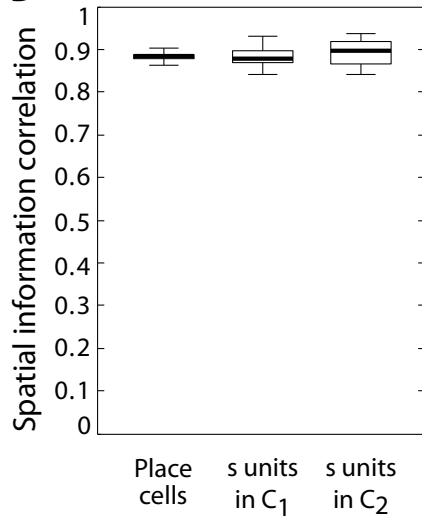

Supplement: Figure S3 — Additional measures of the location selectivity property of neurons in and neurons in . (A) Left: sparseness of single cell responses as measured by their lifetime kurtosis. The larger the kurtosis is, the larger is the sparseness. Right: the size of the receptive field (see Fig. 4B) is anti-correlated to the lifetime kurtosis measure. (B) Left: sparseness of the population place code as measured by the population kurtosis function. Right: the density of receptive fields (see Fig. 5C) is anti-correlated to the population kurtosis measure. (C) The spatial information sparseness –computed as the ratio between population information and the sum of single cell information– demonstrates that the hippocampal place code is redundant in terms of spatial information content. By contrast, although loosing part of the spatial information, the cortical population achieves a better coding, maximizing the contribution of each unit to the population code, particularly for the population. (D) Spatial information Pearson correlation. Expectedly, the way spatial information is encoded by neurons firing rates is not different between the three populations: they all have their surprise information strongly correlated with the strength of the discharge activity. (PDF) [file pcbi.1002045.s003.pdf]

A

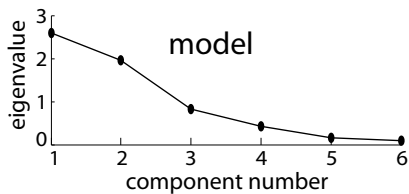

B

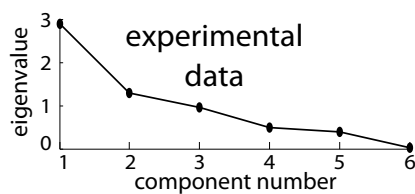

C

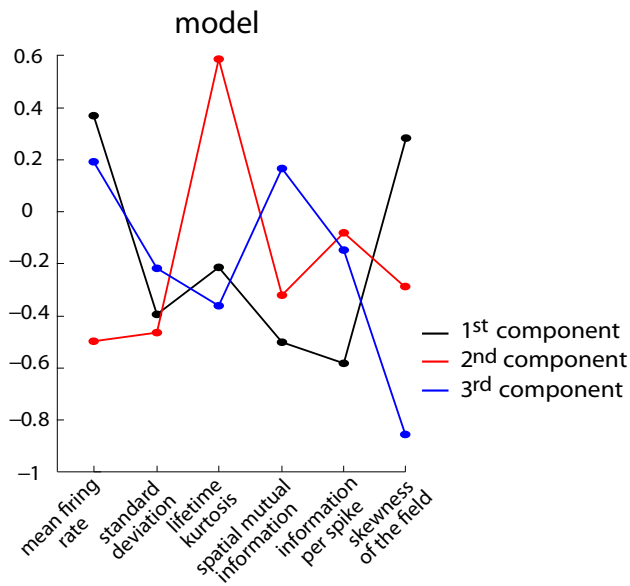

D

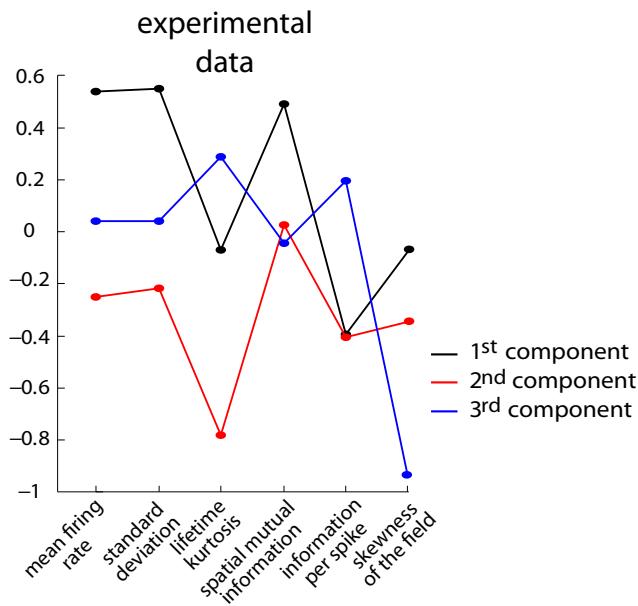

Supplement: Figure S4 — Principal component analysis of simulated (left) and real (right) neuronal activities. Eigenvalues (top) and structure of the principal components (bottom). (PDF) [file pcbi.1002045.s004.pdf]

A

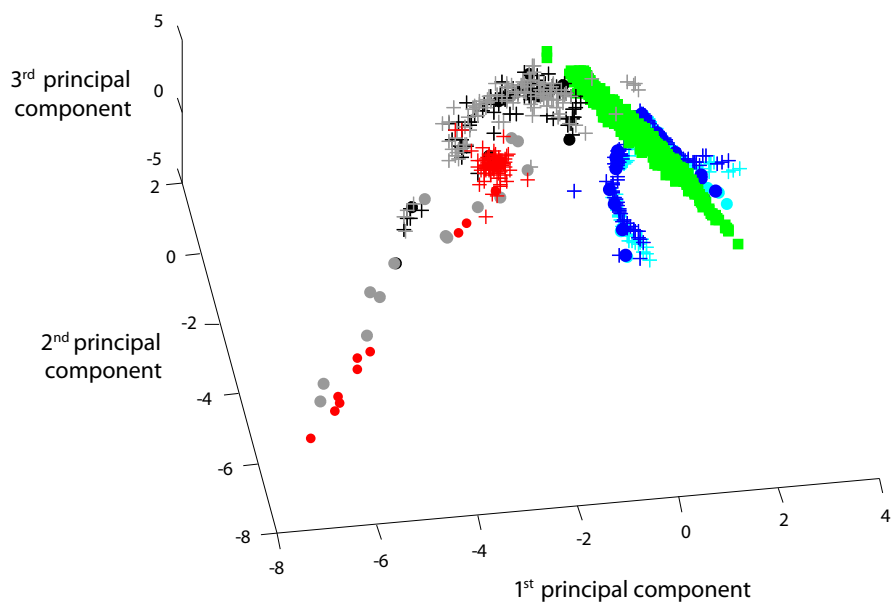

B

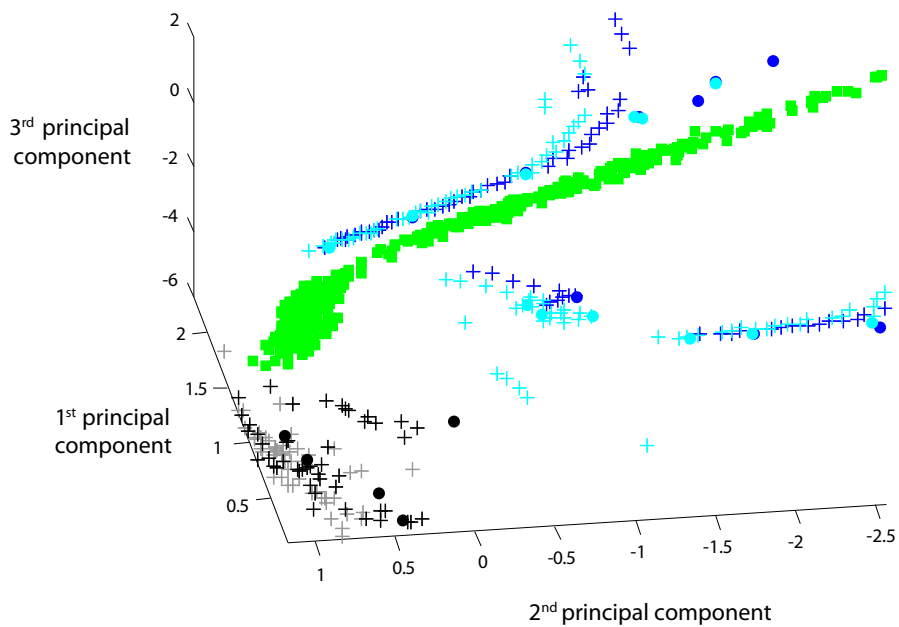

Supplement: Figure S5 — Principal component analysis (PCA) of simulated neuronal activity. Comparison between model and random population activities. Two different views of the same three-dimensional PCA space are shown (A and B, respectively). The size of the original data set used for the analysis reported on Fig. 9 was doubled by adding a population of Poisson neurons. The distribution of the mean firing rates over the original data set was fitted by the distribution of the mean firing rates computed over the population of Poisson neurons. (PDF) [file pcbi.1002045.s005.pdf]

A

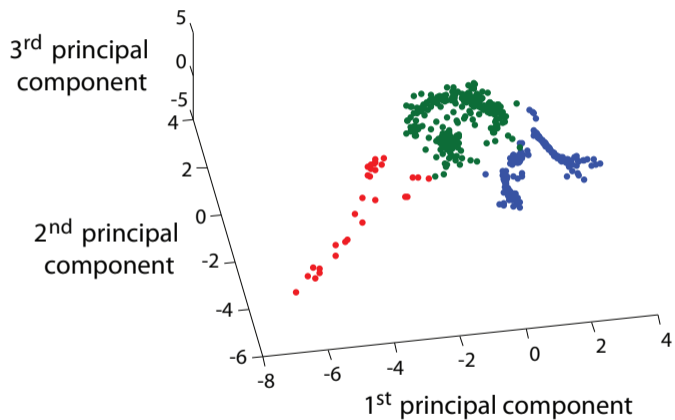

B

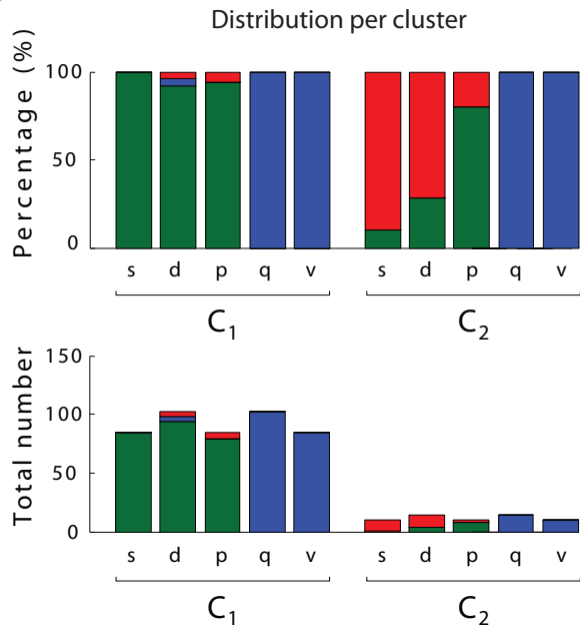

Supplement: Figure S6 — Principal component analysis (PCA) and unsupervised clustering of simulated neuronal activities. (A) Clustering of model activities within the PCA space (first three principal components). (B) Distribution of neural populations for each cluster (top: percentages; bottom: absolute counts). (PDF) [file pcbi.1002045.s006.pdf]

A

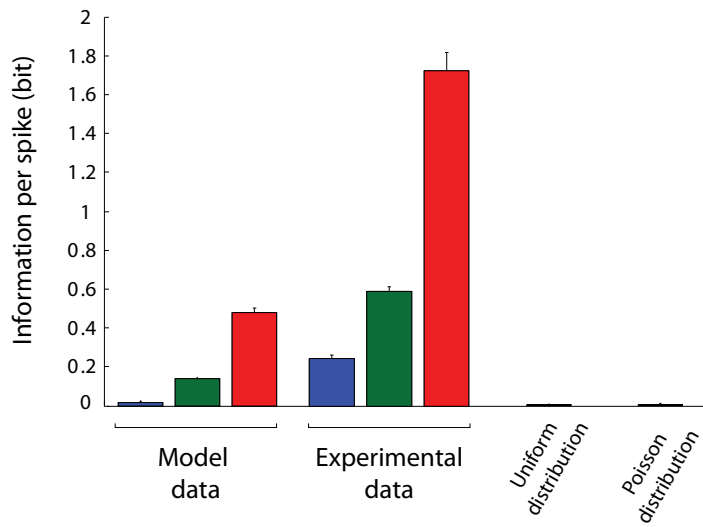

B

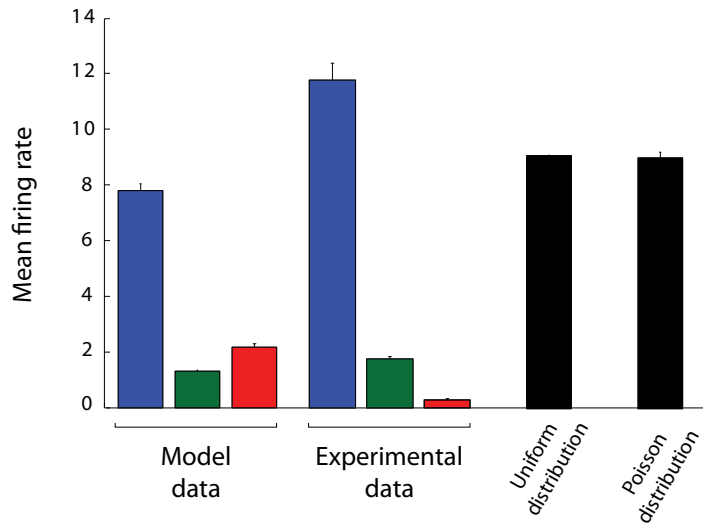

C

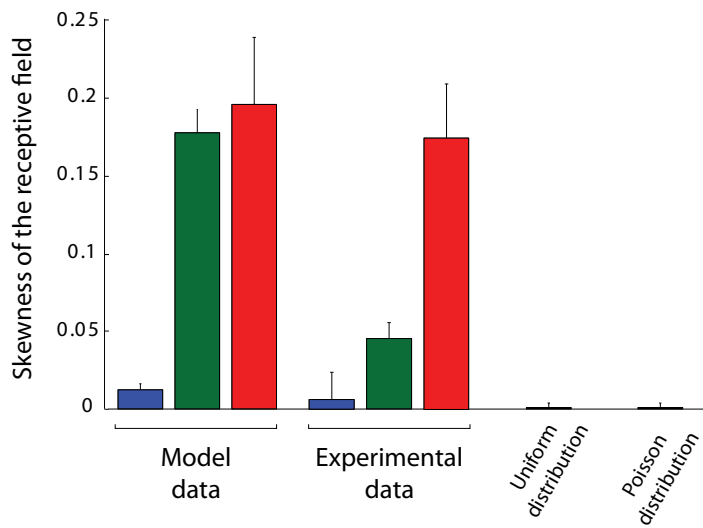

Supplement: Figure S7 — Principal component analysis (PCA): control analysis for the comparison between experimental and model data shown in Fig. 10. (A) Information per spike computed for model rescaled data, experimental data and two random neuron populations (formed by neurons each). Model data in this figure were obtained by rescaling the mean firing rates of model neurons from to , where denoting the maximum mean firing rate observed in experimental data. The first random population, called “Uniform distribution”, consisted of neurons discharging between and according to a uniform distribution. The second control population, called “Poisson distribution”, generated random activities following Poisson distributions with parameters (i.e. means) drawn from an uniform distribution between 0 and R. As expected, the two random populations exhibited extremely weak information content and could not explain the high spatial information found in experimental data. (B) Mean firing rate for the same four sets of data. This figure provides a mere empirical validation of the process used to draw random neural responses with mean firing rates within the range of those of experimental and rescaled model data. (C) Skewness of the receptive field for the same four sets of data. The random neural populations did not have asymmetrical deformation of their response profiles, and thus could not explain the values observed experimentally. (PDF) [file pcbi.1002045.s007.pdf]
