# Supplementary material for: Spatial Learning and Action Planning in a Prefrontal Cortical Network Model
Source: PLoS Comput Biol. 2011 May 19;7(5):e1002045. doi: 10.1371/journal.pcbi.1002045 (PMC3098199; doi:10.1371/journal.pcbi.1002045)
Supplement: Text S1 — Detailed account of the model. This document provides equations and parameter settings related to the column model, the connectivity layout and the learning rules shaping the dynamics of the network. (PDF) [file pcbi.1002045.s008.pdf]

Supplementary Text S1: Columnar model, connectivity layout, and learning rules

Neuronal model

The elementary computational units of the model are firing-rate neurons i, whose mean discharge $r_i \in [0,1]$ is given by:

$$r_i(t) = f\Big(V_i(t) \pm \eta\Big) \tag{1}$$

where $V_i(t)$ is the membrane potential at time t, f is the transfer function (when not specified $f_0(x) = x$), and η denotes random noise uniformly drawn from $[0, 0.1]$. The membrane potential V_i varies according to:

$$\tau_i \cdot \frac{dV_i(t)}{dt} = -V_i(t) + I_i(t) \tag{2}$$

where $\tau_i = 10$ ms is the membrane time constant, and $I_i(t)$ is the input synaptic drive. Eq. 2 is integrated by using a time step $\Delta_t = 1$ ms. For a given neuron i receiving inputs from an afferent population J, the synaptic drive $I_i(t)$ is taken as:

$$I_i(t) = \max_{j \in J} \{w_{ij} \cdot r_j(t)\} \tag{3}$$

where $w_{ij} \in [0,1]$ indicates the synaptic weight of the projection from the presynaptic neuron j to the postsynaptic neuron i. See [1, 2] for plausible neuronal implementations of max operators.

Column network connectivity

Every column of the model assembles three units s, p, v plus a population of minicolumns, each composed of two units q and d (Fig. 1B).

Intra-column connectivity layout

Neurons s, p respectively in layer V-VI and II-III, project onto units d of layer V-VI by means of one-to-all non-plastic synapses. Each neuron q in layer II-III sends a constant one-to-one projection to the corresponding d neuron in the same minicolumn. Each neuron v in layer II-III receives all-to-one projections from neurons q in layer II-III of the column (Fig. 1B).

The activity of neurons s, p, v, q varies according to Eq. 3. The discharge of every neuron q induces a multiplicative effect on $s \to d$ and $p \to d$ synapses. The synaptic drive of a d neuron is taken as:

$$I_d(t) = \max\Big\{w_{ds} \cdot r_s(t)\ ,\ w_{dp} \cdot r_p(t)\Big\} \cdot w_{dq} \cdot r_q(t) \tag{4}$$

where $w_{ds} = w_{dp} = w_{dq} = K = 1$ denote the weight matrices of $s \to d$, $p \to d$, and $q \to d$ synapses, respectively.

Inter-column connectivity layout and input-output of the network

Before learning, the connection patterns are general for all columns of the model. They are adapted during the learning process to specialize the network in two subpopulations C_1 and C_2 (Sec. Spatial learning: encoding topological representations). The model network relies on plastic connectivity and considers both sub-cortical projections and cortical collaterals (Fig. 1B):

- Neurons s of the model can receive three types of afferent information: *(i)* direct spatial inputs from hippocampal place (HP) cells via projections w_{sh}; *(ii)* indirect (pre-processed) state-related inputs from other cortical neurons s via collaterals w_{ss}; *(iii)* putative proprioceptive information ϕ encoding changes in motion direction (see below).
- Neurons p receive: *(i)* recurrent projections w_{pp} from other neurons p of the network; *(ii)* collaterals w_{pd} from neurons d of other PFC columns (used to encode forward associations between places).
- Neurons q receive: *(i)* recurrent projections w_{qq} from other neurons q of the network; *(ii)* collaterals w_{qv} from neurons v of the network (used to encode reverse place associations).
- Neurons v receive: *(i)* sub-cortical motivation-dependent signals via projections w_{vm} (used during learning to associate a column to a rewarding location); *(ii)* collaterals w_{vv} from other neurons v of the network.
- Infragranular neurons d form the outputs of the column and project to motor-related areas [3]. During exploration, each neuron d becomes selective to a specific (allocentric) motion direction.

Spatial learning: encoding topological representations

The cortical network starts with weak synaptic weights randomly initialized within $[0, 0.1]$. As exploration proceeds, all plastic projections w_{sh}, w_{qv}, w_{pd}, w_{ss}, w_{pp}, w_{qq}, w_{vm} and w_{vv} are learned to encode topological maps. As shown in Fig. 1A, the cortical network model performs a two-stage processing of state-related information. During spatial learning, a subpopulation C_1 of cortical columns becomes primarily selective to spatial inputs received directly from hippocampal place (HP) cells, whereas another subpopulation C_2 processes state-related inputs from recurrent projections from C_1. For sake of clarity, we first describe spatial learning at the level of C_1 columns and then at the more abstract level encoded by C_2.

State learning in C_1 population (w_{sh})

The state learning scheme reinforcing afferent connections from the hippocampal place cells to s units begins when the place field representation gets stable, that is when the place field density for all places $s \in S$ visited by the simulated animal is above threshold:

$$\forall s \in S \sum_{h \in HP} \mathcal{H}\Big(r_h(s) - \eta\Big) > \zeta \tag{5}$$

where $r_h(s) \in [0, 1]$ is the response of a neuron $h \in HP$ when the animal is visiting the location $s \in S$, η denotes the noise level activity, $\zeta = 6$ and $\mathcal{H}$ is the Heaviside function (i.e. $\mathcal{H}(x) = 1$ if $x > 0$, $\mathcal{H}(x) = 0$ otherwise). In other word, the place field representation is considered stable when at least $\zeta = 6$ place cells are active at any places visited by the animal.

Then, at each location visited by the animal at time t the cortical network is updated if-and-only-if the activity of neurons s of all existing columns is below threshold:

$$\sum_{s \in C_1} \mathcal{H}\big(r_s(t) - \rho\big) = 0 \tag{6}$$

where r_s is the firing rate of neuron $s \in C_1$ and $\rho = 0.3$. If the novelty condition holds (Eq. 6), then a new column becomes selective to that location by potentiating the projections w_{sh} from all active place cells to the neuron s of the column:

$$w_{sh} = \mathcal{H}\big(r_h(t) - \rho\big) \cdot r_h(t) \tag{7}$$

where $r_h(t)$ denotes the firing rates of place cells $h \in$ HP. If the location visited at time t is not novel (i.e. Eq. 6 does not hold), a winner-take-all scheme selects the most active neuron s of the cortical network and an unsupervised Hebbian learning rule regulates the strength of its hippocampal afferents w_{sh} according to:

$$\Delta w_{sh} = \alpha \cdot r_s(t) \cdot \big(r_h(t) - w_{sh}\big) \tag{8}$$

where $\alpha = 0.2$ is the learning rate.

State connectivity learning in C_1 population (w_{pd} and w_{qv})

The model exploits PFC excitatory collaterals [3, 4] to encode the spatial connectivity between places. During exploration, projections w_{pd} and w_{qv} (Fig. 1B) are modified to learn forward and reverse place associations, respectively. Let $c, c' \in C_1$ denote the columns coding for the rat position before and after a state transition, respectively. One minicolumn, i.e. a pair of q and d neurons in c, becomes selective to this transition. In particular, the neuron d is associated to the locomotion orientation taken by the animal to perform the transition. The weight $w_{p'd}$ of the projection from $d \in c$ to $p' \in c'$ and the weight $w_{qv'}$ from $v' \in c'$ to $q \in c$ are modified according to the following LTP/LTD plasticity rule:

$$\Delta w_{p'd} = (1 - \lambda) \cdot (\beta_{LTP} - w_{p'd}) - \lambda \cdot \beta_{LTD} \cdot w_{p'd} \tag{9}$$

where $\beta_{LTP} = 0.9$, $\beta_{LTD} = 0.5$, and the term $(1 - \lambda)$ indicates whether the simulated animal succeeded or failed the transition from c to c' ($\lambda = 0$ or $\lambda = 1$, respectively). If, for example, a new obstacle prevents the simulated rat from achieving a previously learned transition from column c to c', then a depression of the synaptic efficacy $w_{p'd}$ occurs. Note that the learning rule defined by Eq. 9 leads to $w_{pd}, w_{vq} \in [0, \beta_{LTP}]$.

State learning in C_2 population (w_{ss}, w_{pp} and w_{vv})

The example of Fig. S1 illustrates how the cortical network C_2 is established and interconnected to the population C_1 during spatial exploration. Recall that the activity of neurons $s_2 \in C_2$ is driven by both the collateral excitatory inputs from neurons $s_1 \in C_1$, but it also integrates a putative proprioceptive signal ϕ used to encode the probability of steady changes in egocentric locomotion direction. For instance, $\phi \approx 1$ if the animal turns systematically by an angle greater than a threshold of 15° at a given location, whereas $\phi \approx 0$ if the animal goes approximately straight. The signal ϕ modulates the electroresponsiveness of neurons $s_2 \in C_2$. In the model, this modulation is implemented at the level of the transfer function of neurons s_2, which is taken as:

$$f(x) = x \cdot \gamma^{(1-\phi)} \tag{10}$$

with the constant parameter $\gamma = 1.1$. Note that $f(x)$ approximates the identity transfer function $f_0(x)$ when $\phi \approx 1$.

The network C_2 starts with weak connectivity and unsupervised learning modifies the synaptic weight distributions. The novelty condition to update the C_2 network is slightly different from Eq. 6 because it takes into account both the activity of units $s_2 \in C_2$ and the proprioceptive signal ϕ:

$$\phi(t) + \sum_{s_2 \in C_2} \mathcal{H}\big(r_{s_2}(t) - \rho\big) = 0 \tag{11}$$

If Eq. 11 holds, a new column of C_2 becomes interconnected to the most active column of the network C_1 as follows. Let $s_1 \in C_1$ be the most active unit when the novelty condition (Eq. 11) occurs, and let $s_2 \in C_2$ indicate the state neuron of the newly recruited column in C_2 (Fig. S1). The $s_1 \to s_2$ projection is potentiated by:

$$w_{s_2 s_1} = \mathcal{H}\big(r_{s_1} - \rho\big) \cdot r_{s_1} \tag{12}$$

At each time step t, the following learning scheme shapes the interconnections between the most active column in C_1 and the most active column in the population C_2:

$$\Delta w_{s_2 s_1} = \eta \cdot r_{s_1} \cdot r_{s_2} \cdot \mathcal{H}\big(r_{s_1} - w_{s_2 s_1}\big) \cdot (1 - \phi) \tag{13}$$

$$\Delta w_{v_1 v_2} = 1 - w_{v_1 v_2} \tag{14}$$

$$\Delta w_{p_1 p_2} = 1 - w_{p_1 p_2} \tag{15}$$

with $\eta = 0.6$. A consequence of this encoding scheme is that all C_1 columns that are sequentially activated when the simulated animal moves along a straight path (e.g. an alley) tend to be interconnected to the same column in C_2.

The activity of neurons $v_2, p_2 \in C_2$ influences the discharge of neurons $v_1, p_1 \in C_1$ through recurrent connections $w_{v_1 v_2}, w_{p_1 p_2}$, respectively. In the model, this is achieved by modulating the transfer function of units $v_1 \in C_1$ as follows (the same holds for the transfer function of units $p_1 \in C_1$):

$$f(x) = x \cdot \gamma^{\psi} \tag{16}$$

where γ is a constant factor set to 1.1 and $\psi = \mathcal{H}\big(\max_{v_2 \in C_2} \{w_{v_1 v_2} \cdot r_{v_2}\}\big)$. The term ψ allows the activity of neurons $v_1, p_1 \in C_1$ to be enhanced in the presence of a discharge of neurons $v_2, p_2 \in C_2$, respectively. By contrast, $f(x)$ reduces to $f_0(x)$ when no activity from $v_2, p_2 \in C_2$ occurs.

State connectivity learning in C_2 population (w_{pd}, w_{qv}, w_{pp} and w_{qq})

After learning, transitions in the C_2 state-space representation are likely to map steady discontinuities in the environment structure (e.g. a L-turn in an alley of a maze). The example of Fig. S1 shows how recurrent projections w_{qv}, w_{pd}, w_{pp}, and w_{qq} are updated when a state transition occurs in the C_2 representation:

- Let $c_2, c_2' \in C_2$ be the columns encoding the states before and after a transition, respectively.
- Let $(q_2, d_2) \in c_2$ be the minicolumn selective for the $c_2 \to c_2'$ transition.
- Let c_1, c_1' be the columns of C_1 that are active before and after the transition, respectively.
- Let $(q_1, d_1) \in c_1$ be the minicolumn selective for the $c_1 \to c_1'$ transition.

At each time step t the interconnectivity between these units is updated according to:

$$\Delta w_{p_2' d_2} = \frac{w_{p_2' p_1'} \cdot r_{p_1'}}{r_{d_2}} - w_{p_2' d_2} \tag{17}$$

$$\Delta w_{q_2 v_2'} = \frac{w_{q_2 q_1} \cdot r_{q_1}}{r_{v_2'}} - w_{q_2 v_2'} \tag{18}$$

$$\Delta w_{p_2' p_1'} = 1 - w_{p_2' p_1'} \tag{19}$$

$$\Delta w_{q_2 q_1} = 1 - w_{q_2 q_1} \tag{20}$$

This mechanism allows the C_2 network to adapt its topology while accounting for the goal-distance information encoded by neurons C_1. Neuron $q_2 \in C_2$ will mirror the activity of $q_1 \in C_1$, whereas $p'_2 \in C_2$ will mirror $p'_1 \in C_1$. As a consequence, the information propagated at the level of the C_1 network will also be available in C_2. Thus, planning (see below) can be consistently achieved in parallel by C_1 and C_2 based on a bidirectional flow of information between these two cortical populations.

Exploiting the topological representation for navigation planning

Fig. S2 illustrates a simple example of activation diffusion process mediated by the columnar network model during planning. A putative motivation signal first elicits the activity of neurons v in the columns of C_1 and C_2 associated to the goal location (Fig. S2A). The reward-based activity of neurons v is then back-propagated through the reverse state associations encoded by collaterals $w_{vq} \in C_1, C_2$. Each synaptic relay along the neural pathway formed by w_{vq} projections attenuates the back-propagating activity ($w_{vq} < 1$). Thus, the activation diffusion mechanism produces an exponential decrease of the intensity of the goal signal that propagates through the network of columns. It is worth noting that the recurrent dynamics induced by $w_{v_1 v_2}$ and $w_{q_2 q_1}$ increases the time constant of the exponentially decaying propagation (Eqs. 16 and 17). For example, after 10 synaptic relays the activity of a neuron $v \in C_1$ would be $r_v \approx 0.35$ without recurrent dynamics vs. $r_v \approx 0.9$ with the C_2 modulation (Fig. S2B). Hence, the goal-dependent signal can spread over a larger number of columns before reaching the critical level of neuronal noise.

Since the receptive fields of C_1 columns tend to be evenly distributed, the intensity of the goal signal at a given place encodes the distance to the rewarding location. The learning rule implemented for collateral weights in C_2 (Eq. 17) allows this distance-to-goal coding property to be conserved at the level of the C_2 population.

The activity of d neurons integrates this reverse activity flow with the current state in both C_1 and C_2 populations (Eq. 4). In particular, the occurrence of the q input is a necessary condition for a neuron d to fire. In the presence of the q input, either the hippocampal signal relayed by the neuron s or the cortical input transmitted by neuron p is sufficient to trigger the discharge of a unit d (Eq. 4). When the back-propagated goal signal reaches the column selective for the current position, the coincidence of the state-related hippocampal input conveyed by s neurons and the goal-related input transmitted by q neurons activates the neuron d, which in turns triggers the forward propagation of a pathway signal through projections w_{pd}. The activity of d neurons also conveys distance-to-reward information (because d neurons are partially driven by q neurons). Thus, at each step of the forward propagation, the motor action associated to the most active neuron d can be selected and the sequence of actions from the current position to the goal can be iteratively readout (Fig. S2A).

References

1. Riesenhuber M, Poggio T (1999) Hierarchical models of object recognition in cortex. Nat Neurosci 2: 1019–1025.

2. Yu AJ, Giese MA, Poggio TA (2002) Biophysiologically plausible implementations of the maximum operation. Neural Comput 14: 2857–2881.

3. Mountcastle VB (1997) The columnar organization of the neocortex. Brain 120: 701–722.

4. Lewis DA, Melchitzky DS, Burgos G (2002) Specificity in the functional architecture of primate prefrontal cortex. J Neurocytol 31: 265–276.
