# Supplementary material for: Spatial Learning and Action Planning in a Prefrontal Cortical Network Model
Source: PLoS Comput Biol. 2011 May 19;7(5):e1002045. doi: 10.1371/journal.pcbi.1002045 (PMC3098199; doi:10.1371/journal.pcbi.1002045)
Supplement: Text S2 — Statistical analyses of neural activities. This document provides a description of the set of statistical measures used to characterize the model neural code. (PDF) [file pcbi.1002045.s009.pdf]

# Supplementary Text S2: Statistical analyses of neural activities

In order to quantify spatial-related correlates of neural activity, the continuous two-dimensional input space was discretized by a grid of 5 x 5 cm square regions (pixels). Let $S$ denote the set of stimuli, i.e. the set of locations $s$ visited by the simulated animal while solving the task.

## Place field area

For each neuron, the mean firing rate associated to each pixel $s$ was computed by dividing the spike count associated to $s$ by the time spent by animal in $s$. The size of a receptive (place) field was then taken as the number of adjacent pixels with a firing rate above the grand mean rate —i.e. total spike count divided by the total time spent moving in the maze— plus the standard deviation (similarly to [1, 2]).

## Spatial density of receptive fields

To assess the redundancy level of a spatial code —i.e. the average number of units encoding a spatial location $s \in S$— the following density measure was used:

$$D_S = \left\langle \sum_{j \in J} \mathcal{H}\Big(r_j(s) - \eta\Big) \right\rangle_{s \in S} \qquad (1)$$

where $r_j(s)$ is the response of a neuron $j \in J$ when the animal is visiting the location $s \in S$, $\eta$ denotes the noise level activity, and $\mathcal{H}$ is the Heaviside function.

## Sparseness and shape of the spatial code

The *kurtosis* function —i.e. the normalized fourth central moment of a probability distribution and estimates its degree of peakedness— was applied to quantify the sparseness across the neural population and across time (for single neurons) [3].

### Population kurtosis

$$K_P(s) = \left\langle \left[ \frac{r_j(s) - \bar{r}_J(s)}{\sigma_J(s)} \right]^4 \right\rangle_{j \in J} - 3 \qquad (2)$$

with $\bar{r}_J(s)$ and $\sigma_J(s)$ representing the mean and the standard deviation of the population activity distribution for a given stimulus $s$, respectively, was used to estimate how many neurons $j$ in a population $J$ were, on average, simultaneously responding to a stimulus $s$.

### Single cell lifetime kurtosis

$$K_L(j) = \left\langle \left[ \frac{r_j(s) - \bar{r}_j}{\sigma_j} \right]^4 \right\rangle_{s \in S} - 3 \tag{3}$$

with $\bar{r}_j$ and $\sigma_j$ being the mean and the standard deviation of the cell response $r_j$ to the set of stimuli $S$, respectively, was employed to assess how rarely over time a neuron $j$ responded to stimuli $S$.

### Single cell skewness

In order to quantify the degree of asymmetry of spatial receptive fields, we used the skewness measure, defined as the third moment of the place field firing rate distribution [4]. Given a random variable $X_j$ with $N$ samples whose distribution matches the shape of the receptive field of the cell $j$, the skewness $S_k(j)$ is:

$$S_k(j) = \left\langle \left[ \frac{X_j(i) - \bar{X}_j}{\sigma_{X_j}} \right]^3 \right\rangle_{i \in N} \tag{4}$$

## Spatial information content of the spatial code

An information theoretical analysis quantified how much information the neural responses $r \in R$ conveyed about spatial locations $s \in S$. *Shannon mutual information* $\mathrm{I}(R;S)$ [5,6] between neural responses $R$ and spatial locations $S$ was computed:

$$I(R;S) = \sum_{s \in S} p(s) \sum_{r \in R} p(r|s) \cdot \log_2 \left( \frac{p(r|s)}{p(r)} \right) = \sum_{s \in S} p(s) \cdot I(R;s) \tag{5}$$

where $p(r|s)$ indicates the conditional probability of recording a response $r$ while having the simulated rat visiting a region $s$; $p(s)$ the a priori probability computed as the ratio between time spent at place $s$ and the total time; $p(r) = \sum_{s \in S} p(s) \cdot p(r|s)$ the marginal probability of observing a neural response $r$; and $I(R;s)$ is the *stimulus-specific surprise* [7,8]. The continuous output space of a neuron $R = [0,1]$ was discretized via a binning procedure (bin-width equal to 0.1). A correcting term $C$ was subtracted to mutual information to limit the sampling bias [9]:

$$C = \frac{\sum_s R_s^+ - R^+ - (|S| - 1)}{2N\, ln(2)} \tag{6}$$

where $R_s^+ = \sum_{r \in R} \mathcal{H}(p(r|s))$ denotes the number of response bins in which the occupancy probability $p(r|s) > 0$; $R^+ = \sum_{r \in R} \mathcal{H}(p(r))$ denotes the number of response bins where $p(r) > 0$; $|S|$ is the number of stimuli; $N$ is the number of stimulus-response pairs $(s,r)$.

While the kurtosis measures the shape of a response distribution, the mutual information quantifies the reliability of the neural spatial representation in terms of decoding efficacy. Mutual information was computed considering both the responses of single units $j$, $I_j(R;S)$, and the neural population responses, $I_{pop}(R;S)$. The ratio:

$$I^*(R;S) = \frac{I_{pop}(R;S)}{\sum_{j \in J} I_j(R;S)} \tag{7}$$

was used to measure the "information sparseness" of a population code, or, conversely, the redundancy level of the spatial information content of a neural code.

Finally, for a given neuron $j$, the Pearson correlation coefficient $PC(j)$ between the firing rate $r_j$ and the stimulus-specific surprise $I_j(R;s)$, was computed to measure the degree of localization of the spatial code [8]:

$$PC(j) = \frac{\left\langle \left(I_j(R;s) - \bar{I}_j\right) \cdot \left(r_j(s) - \bar{r}_j\right)\right\rangle_{s\in S}}{\sigma_{I_j} \cdot \sigma_j} \quad (8)$$

with $\bar{I}_j$ and $\sigma_{I_j}$ being, respectively, the mean and the standard deviation of the stimulus-specific surprise of neuron $j$ for the set of stimuli $S$.

Mutual information measures the mean information content over the whole environment, but it does not quantifies the specificity of the neuronal discharges. Thus, we employed an additional measure, namely the information per spike $I_{spike}$ [10] defined for a neuron $j$ as:

$$I_{spike}(j) = \sum_{s\in S} \frac{r_j(s)}{\bar{r}_j} \cdot \log_2\left(\frac{r_j(s)}{\bar{r}_j}\right) \cdot p(s) \quad (9)$$

# Behavioral relevance of neural responses

We estimated the mutual information $I_t(R;F)$ between task-related information (the phase $f \in F$ of the protocol, e.g. "Day 1", "Day 2-14" or "Day 15" period) and the firing activity $r \in R$ of a given neural population:

$$I_t(R;F) = \sum_{f\in F}\sum_{r\in R} p(r,f)\ \log_2\left(\frac{p(r,f)}{p(r)\ p(f)}\right) \quad (10)$$

where $p(r,f)$ is the joint probability of observing a population response $r$ when solving the phase $f$ of the protocol; $p(f)$ indicates the a priori probability computed as the ratio between the length of phase $f$ and the total length of the protocol; and $p(r) = \sum_{f\in F} p(r,f)$ is the marginal probability of observing the response $r \in R$.

# References


1. Muller RU, Kubie JL, Ranck JB (1987) Spatial firing patterns of hippocampal complex-spike cells in a fixed environment. J Neurosci 7: 1935–1950.

2. Hok V, Save E, Lenck-Santini PP, Poucet B (2005) Coding for spatial goals in the prelimbic/infralimbic area of the rat frontal cortex. Proc Natl Acad Sci U S A 102: 4602–4607.

3. Willmore B, Tolhurst DJ (2001) Characterizing the sparseness of neural codes. Netw Comput Neur Syst 12: 255–270.

4. Mehta M (2000) Experience-Dependent asymmetric shape of hippocampal receptive fields. Neuron 25: 707–715.

5. Shannon C (1948) A mathematical theory of communication. Bell Syst Tech J 27: 379–423.

6. Bialek W, Rieke F, de Ruyter van Steveninck RR, Warland D (1991) Reading a neural code. Science 252: 1854.

7. DeWeese M, Meister M (1999) How to measure the information gained from one symbol. Netw Comput Neur Syst 10: 325–340.

8. Bezzi M, Samengo I, Leutgeb S, Mizumori SJ (2001) Measuring information spatial densities. Neural Comput 14: 405–420.

9. Panzeri S, Treves A (1996) Analytical estimates of limited sampling biases in different information measures. Netw Comput Neur Syst 7: 87–107.

10. Skaggs WE, McNaughton BL, Gothard KM, Markus EJ (1992) An information-theoretic approach to deciphering the hippocampal code. In: Adv Neural Inf Process Syst. volume 5, pp. 1030–1037.
